# Supplementary material for: Self-Organization of Embryonic Genetic Oscillators into Spatiotemporal Wave Patterns
Source: Cell. 2016 Feb 11;164(4):656–67. doi: 10.1016/j.cell.2016.01.028 (PMC4752819; doi:10.1016/j.cell.2016.01.028)
Supplement: Document S1. Supplemental Experimental Procedures [file mmc1.pdf]

Cell, Volume *164*

Supplemental Information

Self-Organization of Embryonic Genetic Oscillators into Spatiotemporal Wave Patterns

Charisios D. Tsiairis and Alexander Aulehla

Supplemental Experimental Procedures

Culture and Imaging Conditions

Chambers slides (Lab-Tek) were coated with 50 µg/ml fibronectin in PBS as previously described (Lauschke et al., 2013). The tails of E10.5 mouse embryos were dissected in HEPES-buffered culture medium (DMEM-F12, Cell Culture Technologies, with addition of 0.5 mM glucose, 2 mM glutamine and 1% BSA). Depending on the experimental needs the PSM was cut in halves along the A/P axis, or only the tip of the PSM was excised (Lauschke et al., 2013).The pieces of PSM were combined in groups and dissociated by gentle pipetting. Cells were re-aggregated by centrifugation at room temperature, 400 rcf for 4 min. The pellet of cells was collected and cut in pieces approximately 250µm X 250µm.

When the cell origin was tracked the PSM tissue of E10.5 mouse embryos from $LuVeLu^{het}$;$H2BmCherry^{het}$ X $CD1^{wt}$ crosses were used (Abe et al., 2011; Aulehla et al., 2007). Embryos were sorted according to the fluorescence markers and the PSM pieces were isolated as described. Cells were dissociated in groups according to their origin and mixed as experimental needs dictated.

To inhibit Notch signaling reaggregated PSM cells from the entire PSM were cultured as previously described with culture medium supplemented with 2 µM DAPT (Sigma-Aldrich) (Morohashi et al., 2006).

Imaging was performed with a Zeiss LSM780 laser-scanning microscope. Depending on the recorded fluorophore, reaggregated PSM cells were excited with a Ti:Sapphire Laser (Chameleon-Ultra, Coherent) at a wavelength of 960nm and/or a DPSS 561nm Laser through a 20X Plan Apo objective (numerical aperture 0.8) . A Z-stack of 3–4 planes at a 3-5 µm distance was collected every 10 min.

Mouse strains

The mice used were of the previously described lines R26-H2B-mCherry, LuVeLu, and Mesp2-GFP (Abe et al., 2011; Aulehla et al., 2007; Morimoto et al., 2006). We obtained the Mesp2-GFP line from RIKEN BRC through the National Bio-Resource Project of the MEXT, Japan.

FACS Sorting

For sorting according to the intensity, the dissociated cells were sorted using a MoFlo Legacy High Speed cell sorter (Beckman Coulter Inc.) equipped with a 100 µm nozzle. BD FACSFlow™ sheath (Becton Dickinson GmbH), filtered in-line through a PALL Fluorodyne II filter O.2µm (Pall GmbH), was used as sheath in the acquisition of the samples. Acquisition was triggered on FSC using a

512/15 bandpass (BP) filter and the same filter was used for SSC measurements. Venus fluorescence intensity was measured after filtering through a 545/35 nm bandpass filter, while a second detector measured cellular autofluorescence through a 630/40 nm BP filter. Data was acquired using MoFlo Summit software (Beckman Coulter), while post-acquisition analysis was done with FlowJo 9.2 software (Tree Star, Inc). The sorting was completed within 30 min and the sorted cells were processed as previously described to form aggregates.

Data and Image Analysis

The distance between neighbor foci was measured in FIJI, and MATLAB was used to generate their distribution as well as the comparison to random distribution with the Kolmogorov-Smirnov test. Comparison of mean interfoci distance between different experimental groups was done with Student's t-test in Excel (Microsoft).

The phases of the oscillation in the selected region of interest (ROI) were calculate using the Hilbert transform of the signal as previously described (Lauschke et al., 2013). The phases were unwrapped and differentiated using MATLAB to calculate instantaneous frequency whose inverse is the instantaneous period. The absolute values of the complex numbers generated by Hilbert transform correspond to the collective amplitude inside the specified ROI (Pikovsky et al., 2003). This was normalized to the initial value of the time series to generate a measure of the synchronization inside the ROI.

To compare the phase difference in the signal of different ROIs the extracted phases were subtracted as angles.

Fourier transform was performed using MATLAB for the cosine of the phase signal to identify the dominant frequency in this ROI. The ROI was reduced to a single pixel and Fourier transform was performed for each of them when a spatial map of dominant periods was generated for the sample.

References

Abe, T., Kiyonari, H., Shioi, G., Inoue, K.-I., Nakao, K., Aizawa, S., and Fujimori, T. (2011). Establishment of conditional reporter mouse lines at ROSA26 locus for live cell imaging. Genesis *49*, 579–590.

Aulehla, A., Wiegraebe, W., Baubet, V., Wahl, M.B., Deng, C.-X., Taketo, M.M., Lewandoski, M., and Pourquié, O. (2007). A β-catenin gradient links the clock and wavefront systems in mouse embryo segmentation. Nat Cell Biol *10*, 186–193.

Lauschke, V.M., Tsiairis, C.D., Francois, P., and Aulehla, A. (2013). Scaling of embryonic patterning based on phase-gradient encoding. Nature *493*, 101–105.

Morimoto, M., Kiso, M., Sasaki, N., and Saga, Y. (2006). Cooperative Mesp activity is required for normal somitogenesis along the anterior-posterior axis. Dev. Biol.

300, 687–698.

Morohashi, Y., Kan, T., Tominari, Y., Fuwa, H., Okamura, Y., Watanabe, N., Sato, C., Natsugari, H., Fukuyama, T., Iwatsubo, T., et al. (2006). C-terminal fragment of presenilin is the molecular target of a dipeptidic gamma-secretase-specific inhibitor DAPT (N-[N-(3,5-difluorophenacetyl)-L-alanyl]-S-phenylglycine t-butyl ester). J. Biol. Chem. *281*, 14670–14676.

Pikovsky, A., Rosenblum, M., and Kurths, J. (2003). Synchronization: A Universal Concept in Nonlinear Sciences (Cambridge Nonlinear Science Series) (Cambridge University Press).
