# Supplementary figures and images for: Self-Organization of Embryonic Genetic Oscillators into Spatiotemporal Wave Patterns
Source: Cell. 2016 Feb 11;164(4):656–67. doi: 10.1016/j.cell.2016.01.028 (PMC4752819; doi:10.1016/j.cell.2016.01.028)

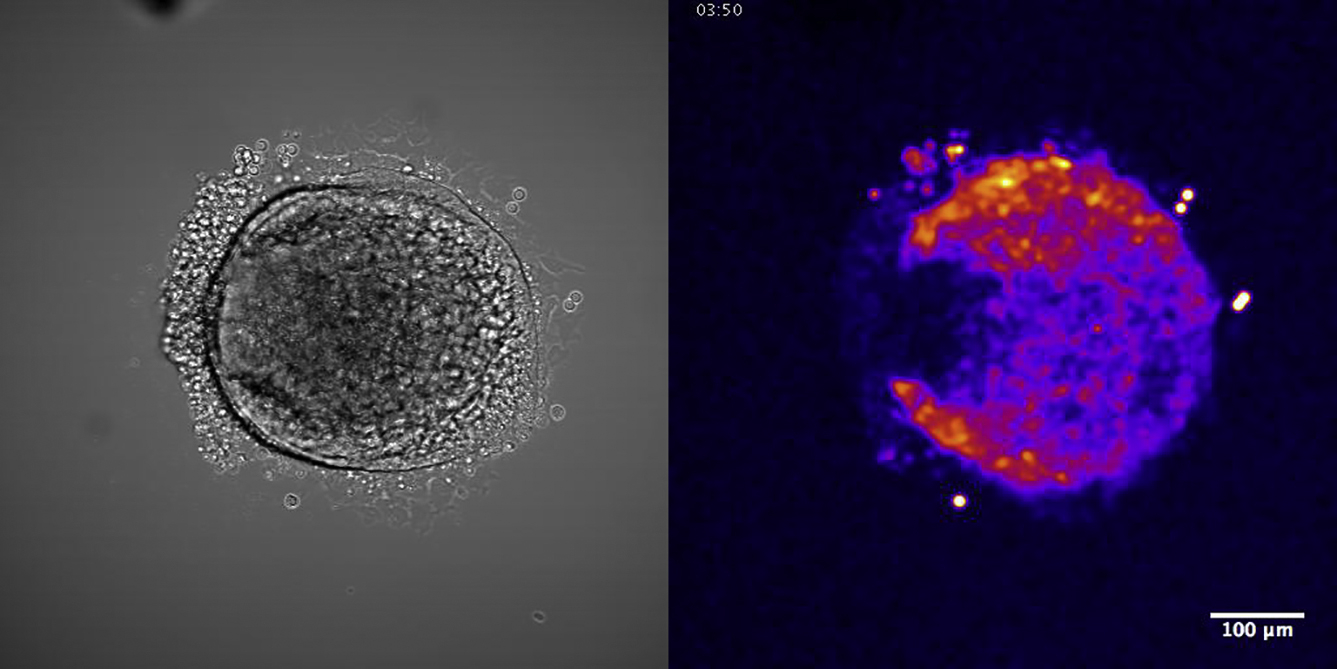

Supplement: Movie S1. Time-Lapse Imaging of 2D Ex Vivo Assay, Related to Figures 1B–1D — Bright field and yellow fluorescent protein (YFP) fluorescence of the LuVeLu transgene are shown. The tail bud of an E10.5 embryo is cultured on a fibronectin-coated coverglass. The coordinated oscillatory expression of the LuVeLu transgene, reflecting rhythmic Notch signaling activity, is detected as a wave of YFP fluorescence sweeping from the center to the periphery of the culture. Time is indicated in hr:min. [file mmc2.jpg]

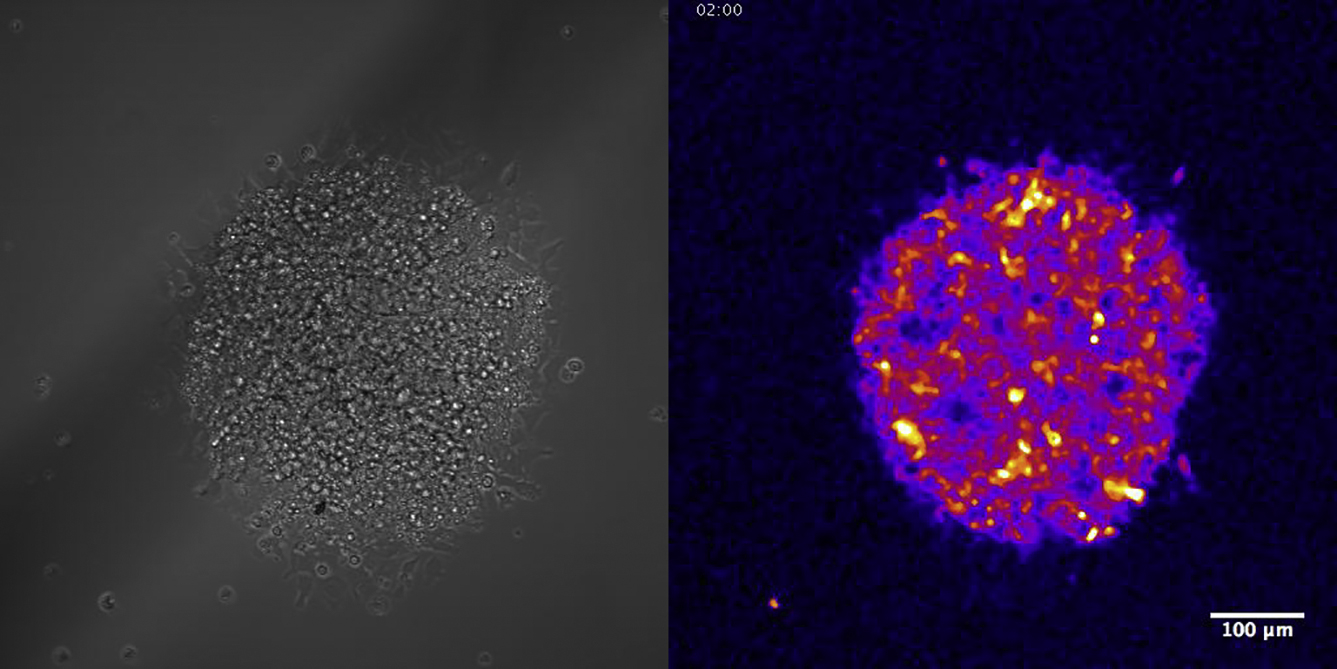

Supplement: Movie S2. Time-Lapse Imaging of Dissociated and then Re-aggregated PSM Cells, Related to Figures 1E–1G — Bright field and YFP fluorescence of the LuVeLu transgene are shown. The movie shows a cell re-aggregate originating from dissociated PSM of six different LuVeLu transgenic E10.5 mouse embryos. The cell aggregate is imaged while it attaches and spreads on a fibronectin-coated coverglass. Over time, several synchronized foci appear showing coordinated oscillation of LuVeLu intensity. In addition, periodic waves become visible within foci, indicating the emergence of spatial and temporal order. Time is indicated in hr:min. [file mmc3.jpg]

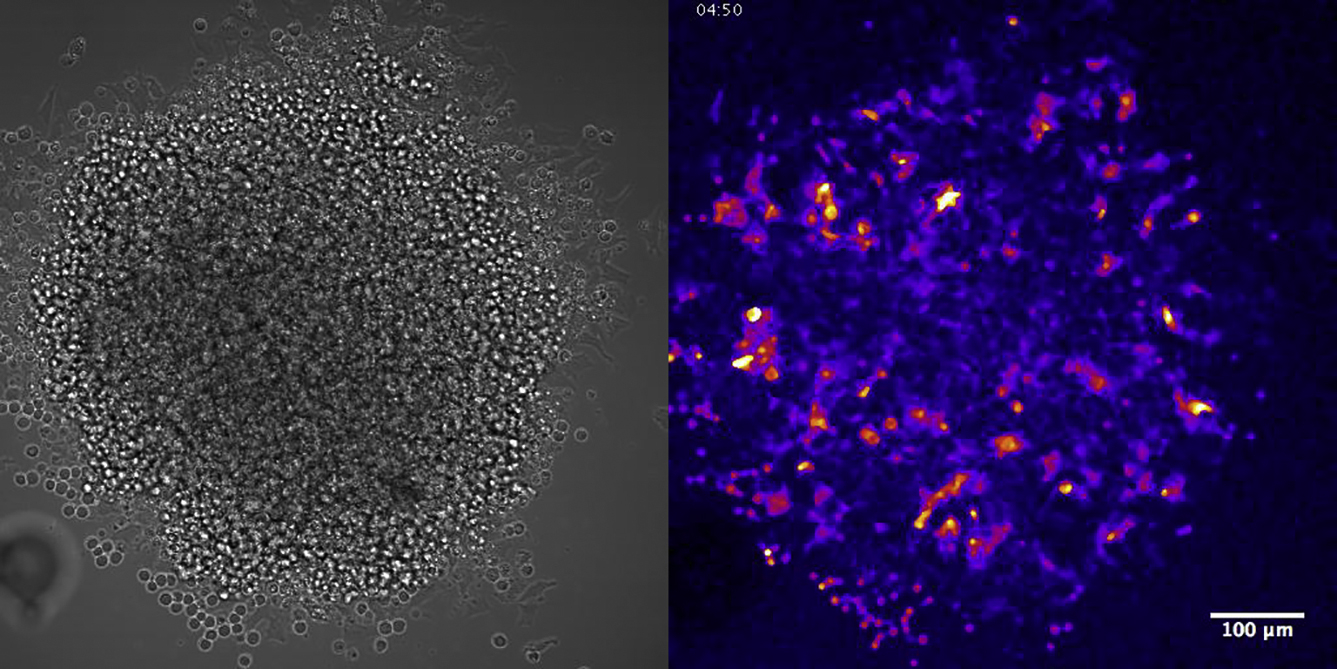

Supplement: Movie S3. Time-Lapse Imaging of Re-aggregated PSM Cells Expressing Mesp2-GFP, Related to Figures 2F and 2G — Re-aggregate contains PSM cells from 6 E10.5 Mesp2-GFP embryos. During the time-lapse recording, GFP expression is detected at the periphery of synchronized foci that form during the culture, indicating that these cells have activated Mesp2 expression and hence initiated the differentiation program leading to segmentation. Over time, the domain that remains negative for GFP shrinks, as cells in the periphery of each ePSM focus differentiate and as no further growth occurs. Time is indicated in hr:min. [file mmc4.jpg]

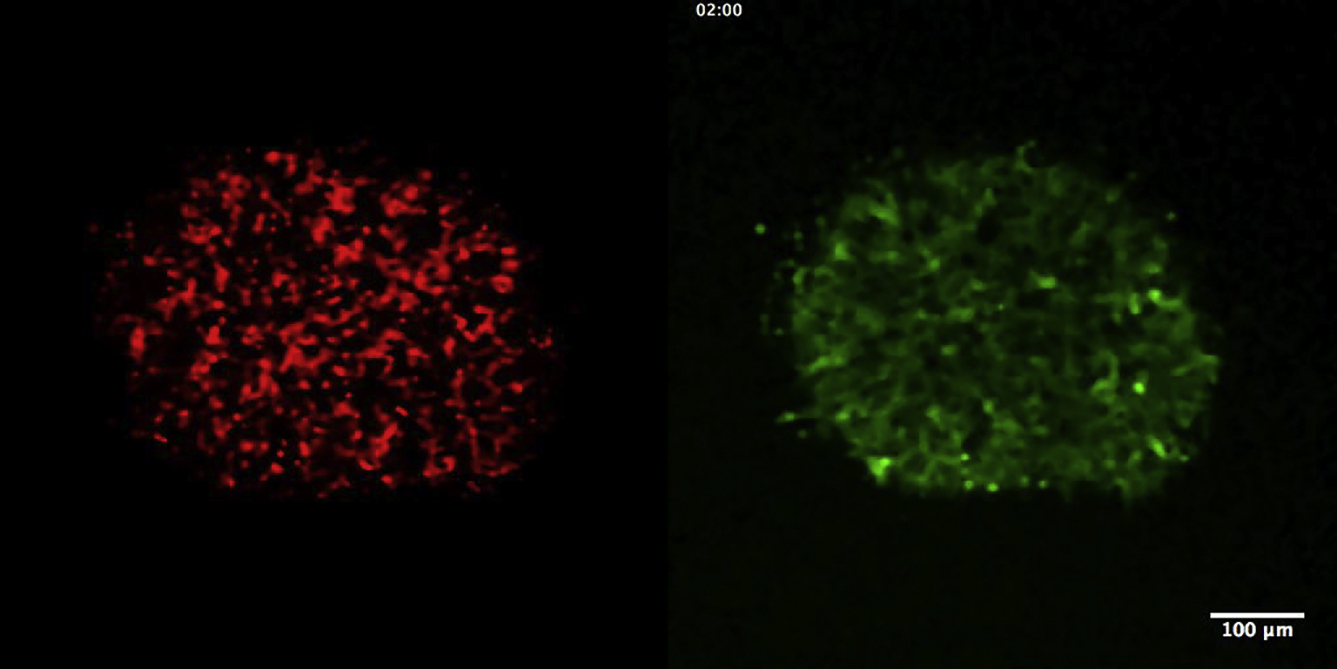

Supplement: Movie S4. Time-Lapse Imaging of Re-aggregated PSM to Analyze Cell Sorting, Related to Figure 3 — H2B-mCherry fluorescence (red) and YFP fluorescence from the LuVeLu transgene (green) are shown. The posterior half of six PSM from embryos positive for H2B-mCherry transgene were dissociated with the anterior half of six PSM from embryos negative for H2B-mCherry and used to form aggregates. All embryos used were positive for the LuVeLu transgene. The formation of foci is seen based on the LuVeLu signal, posterior PSM cells (red) are initially distributed randomly and, over time, sort and localize in foci centers. Time is indicated in hr:min. [file mmc5.jpg]

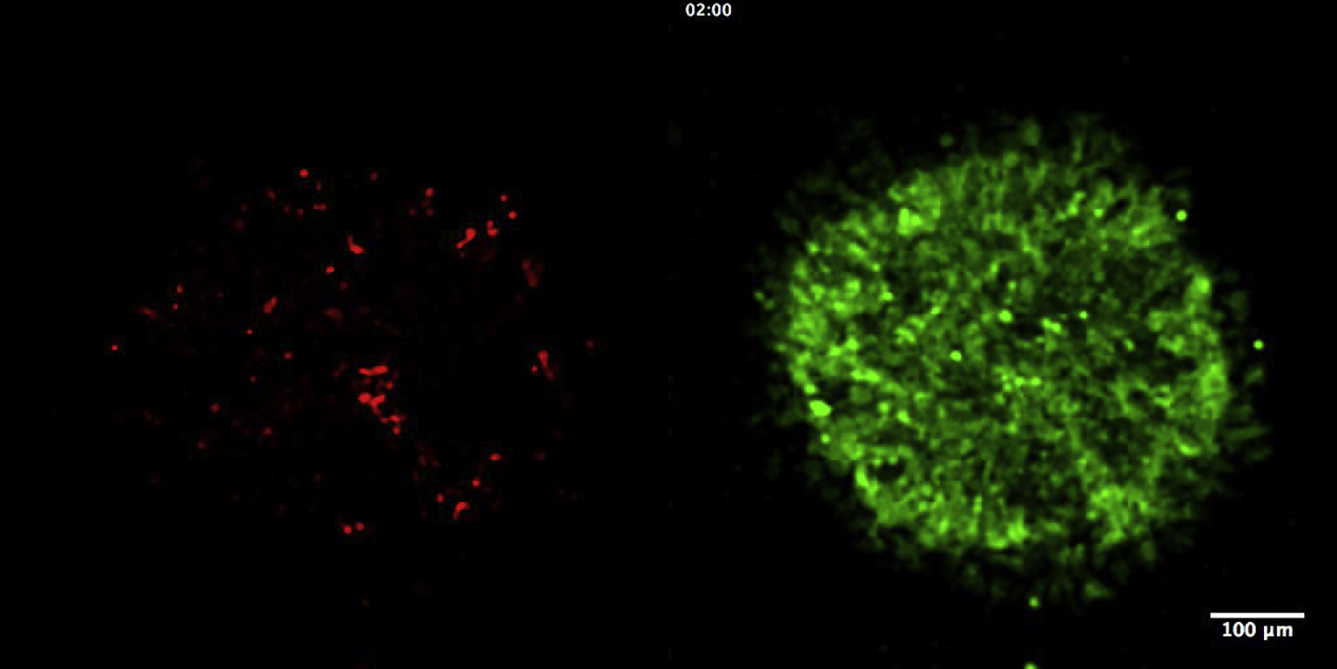

Supplement: Movie S5. Time-Lapse Imaging of Re-aggregated PSM Cells with H2B-mCherry Labeling of Anterior PSM Cells, Related to Figure 7 — All cells carry the LuVeLu transgene, anterior PSM cells (∼1/8 of all cells) are H2B-mCherry positive, and the remaining cells (7/8 of all cells) originate from the posterior tip of the PSM of H2B-mCherry negative samples. Using the red H2B-mCherry label, oscillations of the two populations were quantified separately. Time is indicated in hr:min. [file mmc6.jpg]
