# Supplementary material for: Self-Organization of Embryonic Genetic Oscillators into Spatiotemporal Wave Patterns
Source: Cell. 2016 Feb 11;164(4):656–67. doi: 10.1016/j.cell.2016.01.028 (PMC4752819; doi:10.1016/j.cell.2016.01.028)
Supplement: Document S2. Article plus Supplemental Information [file mmc7.pdf]

Cell

Article

Self-Organization of Embryonic Genetic Oscillators into Spatiotemporal Wave Patterns

Graphical Abstract

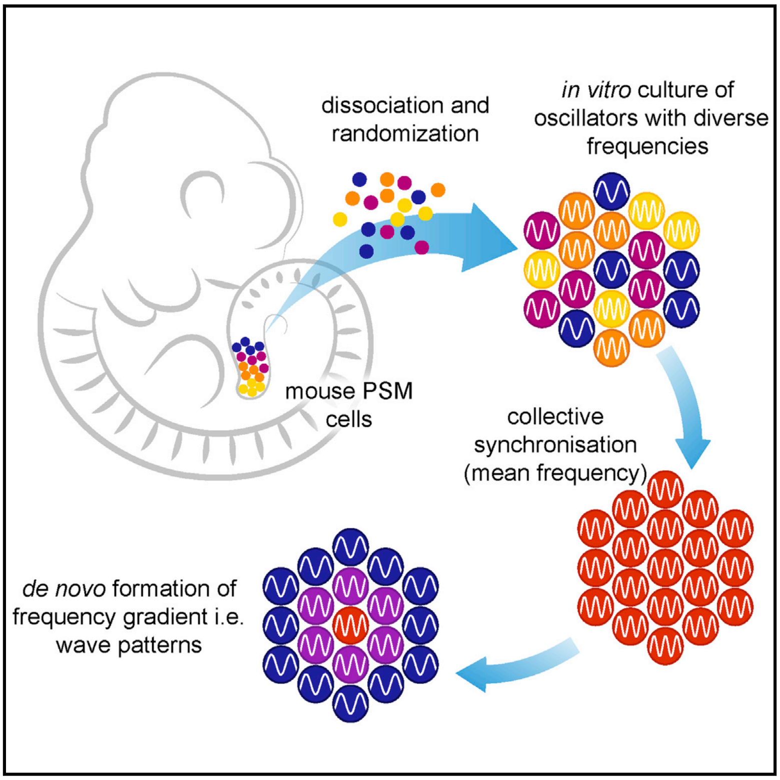

Highlights

- Genetic oscillators in presomitic mesoderm cells generate periodic wave patterns
- A novel in vitro approach addresses the origin of spatiotemporal wave patterns
- Periodic wave patterns emerge de novo upon self-organization of coupled oscillators
- Collective synchronization occurs within ensembles of coupled PSM oscillators

Authors

Charisios D. Tsiairis, Alexander Aulehla

Correspondence

aulehla@embl.de

In Brief

The wave patterns created by genetic oscillators within cells of embryonic presomitic mesoderm (PSM) form in a self-organized manner. When cells are randomized in vitro, the patterns reemerge and the dynamics within each emergent PSM depends on the composition of the ensemble of coupled oscillators, providing evidence for system-level integration.

Tsiairis & Aulehla, 2016, Cell *164*, 656–667
February 11, 2016 ©2016 The Authors
http://dx.doi.org/10.1016/j.cell.2016.01.028

CellPress

Cell

Article

Self-Organization of Embryonic Genetic Oscillators into Spatiotemporal Wave Patterns

Charisios D. Tsiairis[1] and Alexander Aulehla[1,*]
[1]Developmental Biology Unit, European Molecular Biology Laboratory (EMBL), 69117 Heidelberg, Germany
*Correspondence: aulehla@embl.de
http://dx.doi.org/10.1016/j.cell.2016.01.028
This is an open access article under the CC BY license (http://creativecommons.org/licenses/by/4.0/).

SUMMARY

In vertebrate embryos, somites, the precursor of vertebrae, form from the presomitic mesoderm (PSM), which is composed of cells displaying signaling oscillations. Cellular oscillatory activity leads to periodic wave patterns in the PSM. Here, we address the origin of such complex wave patterns. We employed an in vitro randomization and real-time imaging strategy to probe for the ability of cells to generate order from disorder. We found that, after randomization, PSM cells self-organized into several miniature emergent PSM structures (ePSM). Our results show an ordered macroscopic spatial arrangement of ePSM with evidence of an intrinsic length scale. Furthermore, cells actively synchronize oscillations in a Notch-signaling-dependent manner, re-establishing wave-like patterns of gene activity. We demonstrate that PSM cells self-organize by tuning oscillation dynamics in response to surrounding cells, leading to collective synchronization with an average frequency. These findings reveal emergent properties within an ensemble of coupled genetic oscillators.

INTRODUCTION

A fundamental question in biology concerns the origin of ordered patterns. One naturalistic answer that traces the ultimate cause within the living system is self-organization. Self-organized systems achieve order through the properties and interactions of their elements, without the requirement of external guidance. Such systems are abundant at any level of the organization of life (Camazine, 2003). An aggregate of mixed cells from Hydra can self-organize to recreate the entire organism (Gierer et al., 1972). At the organism level, populations of fireflies self-organize and display synchronized flashing (Buck and Buck, 1966). In this case, each animal is an oscillator that adjusts its own rhythm according to the flashing of the neighbors, leading to a common rhythm (Mirollo and Strogatz, 1990). Here, temporal self-organization emerges from the interactions of coupled oscillators.

A genetic, coupled oscillator system functions during embryo development within cells of the presomitic mesoderm (PSM), from which the segmental elements of vertebrates, termed somites, form (Palmeirim et al., 1997). These genetic oscillators involve the periodic activation of several signaling pathways, such as Notch, Fgf and Wnt, with oscillatory activity showing a period matching the rate of somite formation, i.e., 2–3 hr in mouse embryos (Dequéant et al., 2006). Most remarkably, oscillations lead to coherent spatiotemporal wave patterns that sweep through the PSM from posterior to anterior (Aulehla et al., 2008; Masamizu et al., 2006).

Despite several studies addressing the function of spatiotemporal wave patterns (Lauschke et al., 2013; Oginuma et al., 2010; Stauber et al., 2009), it is unclear how these coherent spatiotemporal wave patterns originate and are established in the first place. Molecularly, Notch signaling has been shown to be essential to maintain synchrony between PSM cells, as oscillations drift out of synchrony in both mouse and fish embryos if Notch signaling is disrupted (Delaune et al., 2012; Jiang et al., 2000; Okubo et al., 2012). At the same time, previous experiments have indicated that wave patterns persist largely unperturbed even when the PSM is disrupted or cut into many isolated PSM fragments (Maroto et al., 2005). Combined, these dynamics are therefore commonly described as kinematic waves based on autonomous oscillatory activities (Palmeirim et al., 1997), which are further fine-tuned (via Notch signaling) by cell-cell communication (Herrgen et al., 2010; Horikawa et al., 2006; Masamizu et al., 2006). However, as previous experiments employed PSM in which coherent wave patterns were already present, the role of cell coupling and the potential for self-organization in establishing synchrony and coherent wave patterns remains largely unaddressed.

In this work, we developed experimental approaches to address the principles underlying collective synchronization and the origin of spatiotemporal wave patterns in populations of coupled genetic oscillators.

RESULTS

Randomized PSM Cell Populations Self-Organize in Space and Time

We have previously shown that PSM cells can establish novel coherent spatiotemporal activity patterns in a two-dimensonsal (2D) cell culture context (Figure 1A) (Lauschke et al., 2013). Key

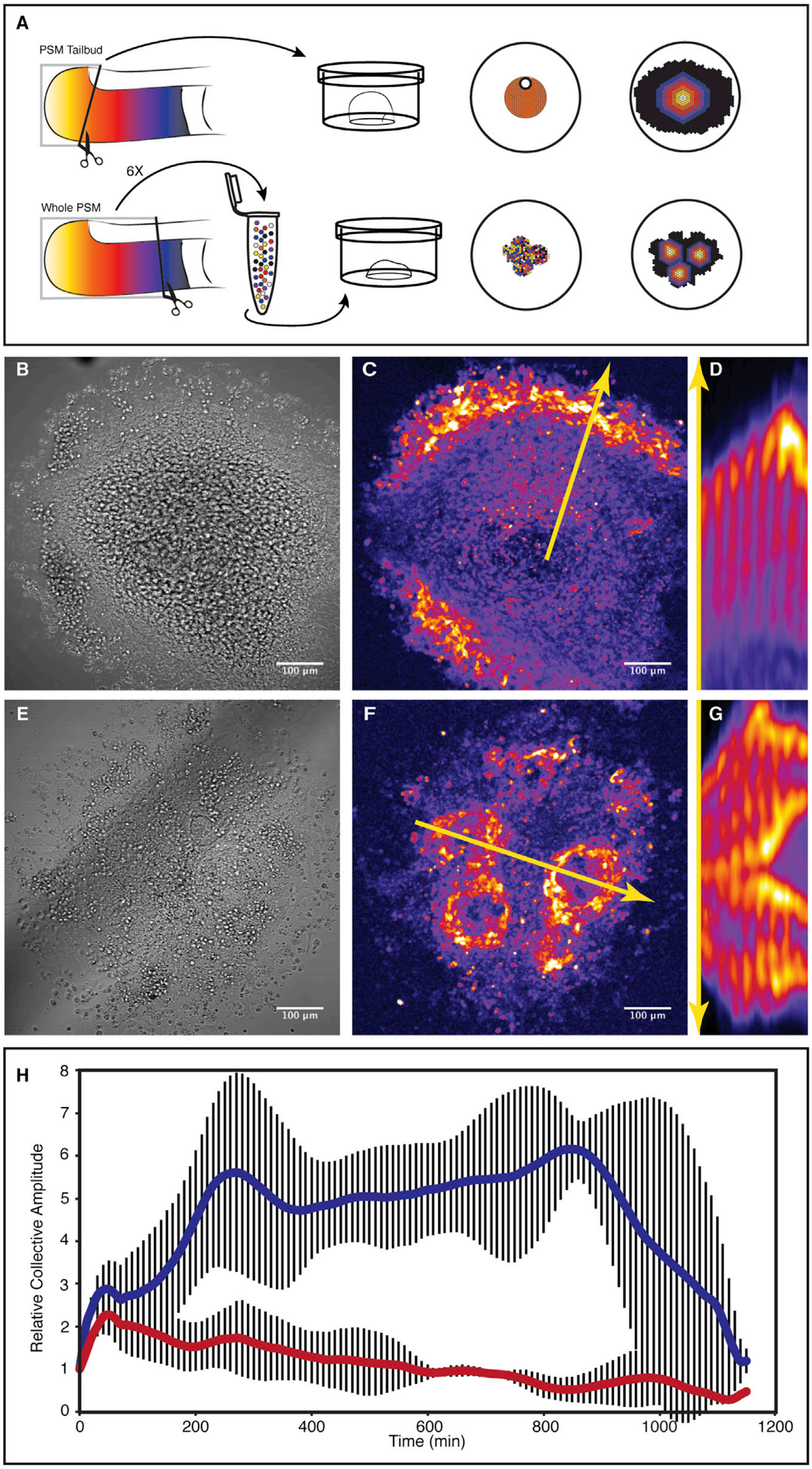

Figure 1. Randomly Mixed PSM Cells Generate Spatiotemporal Patterns in Culture

(A) Schematic representation of the experimental design. A wave of gene activity sweeps the PSM tissue from posterior to anterior. We established a 2D ex vivo primary-cell culture using a posterior PSM slice (tail bud) of a single embryo. Over time, gene activity waves sweep the entire quasi- mPSM culture from the center to the periphery. In contrast, for the randomization assay, the PSM of several embryos (not shown) are dissociated to single cells and these cells are used to generate aggregates of randomly positioned cells. In this system, waves appear from multiple foci.

(B–D) 2D ex vivo assay using intact tail bud explant culture.

(B) Brightfield image of tail bud explant after ~22 hr culture.

(C) Venus fluorescence driven from the lunatic fringe promoter (LuVeLu) is recorded as a wave pattern sweeping from the center to the periphery.

(D) Kymograph along the yellow arrow shown in (C) displays the successive waves of LuVeLu reporter activity in the 2D ex vivo assay.

(E–G) Randomization assay using dissociated and re-aggregated PSM cells.

(E) Brightfield image of re-aggregated PSM cells after ~22 hr of culture.

(F) Periodic LuVeLu expression waves appear in multiple foci within the culture.

(G) Kymograph along the yellow arrow in (F) spanning two such foci reveals coherent spatio-temporal oscillations patterns.

(H) The collective amplitude, as a measure of synchronization within the cell population, is rapidly increasing in control samples (blue line) during randomization assay (± SD for each time point, n = 3). When Notch inhibitor DAPT (2µM) is applied (red line), the collective amplitude remains low (± SD for each time point, n = 3), indicating lack of synchronization between cells.

structural and functional aspects of PSM patterning, including spatiotemporal signaling activities, are recapitulated in the 2D cell culture assay within a monolayer PSM (mPSM) (Figures 1B–1D). However, the question of de novo synchronization of PSM cells could not be addressed in the 2D assay, as the starting conditions preserve cell-cell contacts and hence tissue history. We therefore dissociated the PSM from several embryos into single cells and used the randomized cell suspension to generate dense cell re-aggregates. These were then cultured on fibronectin-coated coverglass, enabling real-time imaging and quantification of signaling activity using a dynamic Notch signaling reporter, LuVeLu (Aulehla et al., 2008). As we used cells from the entire PSM and from several embryos, this setup generated randomized starting conditions, in which all preexisting pattern is lost. Cells encounter random neighbors in terms of oscillation phase, oscillation frequency, and anterior-posterior (A/P) PSM identity (Figure 1A).

We found that after 5–6 hr of culture, cells synchronized and exhibited in-phase oscillations in multiple foci that formed within each re-aggregate. In each of these foci, we identified waves of Notch-signaling activity sweeping the field of cells from the center toward their periphery (Figures 1E–1G). Every successive wave traveled progressively smaller distances as the area swept by waves progressively shrank (Figure 1G). This is reminiscent of the behavior seen in the 2D assay using intact PSM explants and results from the lack of further tissue growth, while cells differentiate and stop oscillations at its periphery (Figure 1D) (Lauschke et al., 2013).

Despite the similarities to the 2D ex vivo culture, in which a single wave origin exists, we find that, in re-aggregates, four to five distinct synchronized foci formed per dissociated PSM (Figures 1C and 1F). The analysis of foci distribution indicated their regular spatial arrangement. Neighboring foci were separated from each other by a minimum distance of 100 μm (Figure S1), with an average of 242 μm ± 70 μm (SD, n = 93). The distribution is significantly different compared to a simulated random localization of foci with similar density (Figure S1). These findings provide evidence for a regulated, self-organized foci patterning process that operates with a characteristic length scale.

To address the molecular mechanism of how de novo synchronization is controlled, we performed self-organization experiments in the presence of DAPT, a chemical inhibitor of Notch signaling (Morohashi et al., 2006). It has previously been reported that "maintenance" of synchronization within the PSM requires Notch signaling (Delaune et al., 2012; Jiang et al., 2000; Okubo et al., 2012). We found that upon DAPT treatment, randomized PSM cells indeed fail to synchronize, as indicated by quantification of the collective amplitude (Figure 1H). Importantly, while no synchrony was evident at the tissue level, single-cell quantifications revealed that, also in DAPT-treated samples, individual cells maintained oscillatory activities, with their amplitude similar to the untreated ones (Figure S2). Thus, de novo synchronization depends on active Notch signaling, in agreement with previous in vivo findings on the maintenance of synchronization.

Self-Organization of Randomized PSM Cells Generates Miniature PSM Patterns

The wave patterns observed in synchronized foci appear similar to those found in 2D ex vivo mPSM assays, despite significant size differences (Figures 1C and 1F). To further analyze this similarity at the molecular level, we examined the expression of PSM markers within the foci. We found expression of *T* (*brachyury*), a marker for posterior mesoderm and PSM (Yamaguchi et al., 1999), to be highly expressed at the center of each newly formed focus (Figures 2A and 2B). In addition, we found that nuclear β-catenin levels, a hallmark of active Wnt signaling, showed a graded distribution within each focus, peaking in the center and decreasing toward its periphery (Figures 2C–2E). This is reminiscent of a Wnt-signaling/β-catenin protein gradient found in the PSM in vivo (Aulehla et al., 2008) and also within the ex vivo mPSM (Lauschke et al., 2013). Finally, we found that over time, *Mesp2*, a key regulator of somite formation (Saga et al., 1997), was upregulated in the periphery of foci (Figures 2F and 2G), indicating that once oscillatory activity ceases, the molecular program of segment formation is initiated. Hence, self-organized foci recapitulate spatiotemporal organization of in vivo PSM. Combined, this molecular analysis indicates that foci represent miniature PSM (that we term emerging PSM [ePSM]) that form spontaneously upon randomization and re-aggregation.

A/P Differences within the Randomized Cell Population Are Not Required to Initiate Self-Organization

To address further the mechanism underlying the emergence of patterns after randomization, we analyzed to which extent cell sorting based on the original PSM position contributes to self-organization. It is known that, within the PSM, cell-adhesion molecules show graded distribution from posterior to anterior (Duband et al., 1987), which can drive cell sorting. Indeed, we found that cells are sorted during culture of re-aggregated PSM cells, with posterior and anterior PSM cells enriched at the center and periphery of each self-organized focus, respectively (Figures 3A–3C). Thus, when cells from the entire PSM are used, self-organization is accompanied by cell sorting according to their original axial position.

To address if this cell sorting according to A/P origin is required for self-organization, we modified the experimental setup and used only cells from the very posterior PSM/tail bud for re-aggregation (Figure 3D). In this case, all cells used for randomization originate from a very similar axial position with minimum differences in A/P-dependent properties, including adhesion. Strikingly, even in this modified experimental setup, we find that oscillating foci appear and that their pattern and spatial arrangement is unaltered compared to the experiments when cells from the entire PSM were used (Figures 3E–3H). Based on these findings, we conclude that, while cell sorting occurs after re-aggregation if cells differ in A/P origin, this cell sorting is per se not required to initiate synchronization, as self-organization of PSM cells occurs even when re-aggregated cells do not show A/P differences.

Self-Organization of PSM Cells into Oscillatory Notch-Activity Wave Patterns

We next analyzed the temporal organization of PSM cells after randomization. To this end, we quantified oscillatory gene activities in real-time experiments and found highly synchronized oscillations within each focus (Figures 4A and 4B). Interestingly, we found that different foci within a single re-aggregate were all

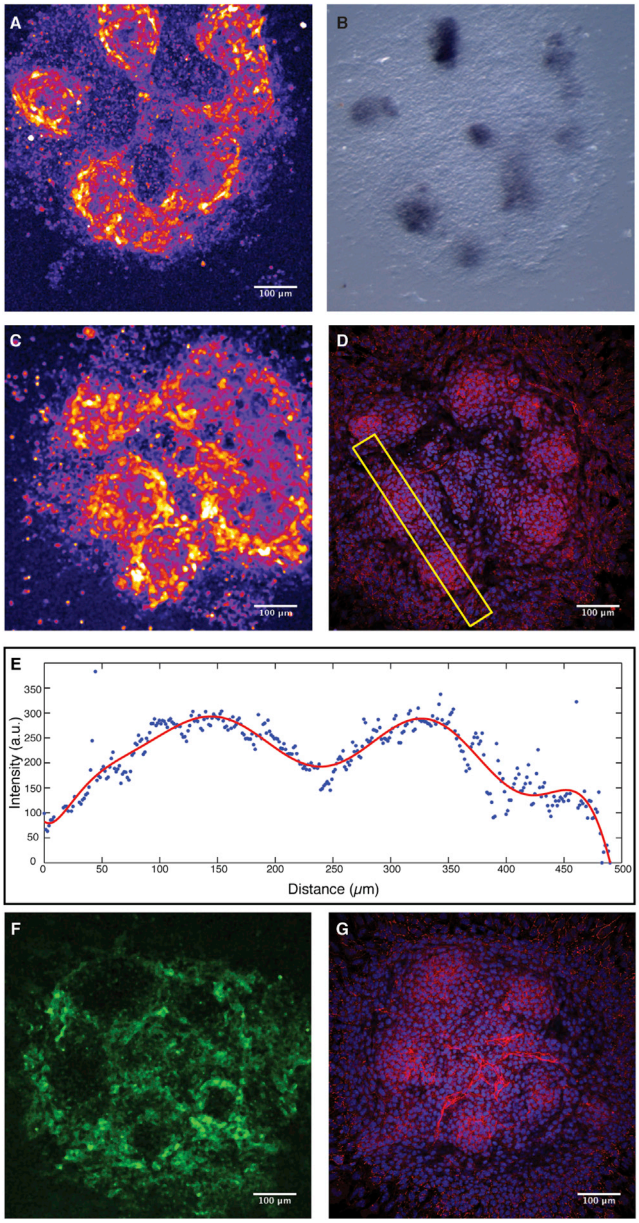

Figure 2. Synchronized Foci in Randomization Assay Resemble Miniature, ePSM

(A) Multiple foci are visible after randomization and ~22 hr of culture using the LuVeLu reporter readout.

(B) In situ hybridization on the same sample shown in (A) reveals mRNA expression of posterior PSM marker *brachyury/T* within the central region of each focus.

(C–D) Foci (LuVeLu readout in C) show high β-catenin protein levels (D). Immunostaining for β-catenin (red) on sample shown in (C) and nuclei (blue) labeled with DAPI.

(E) Quantification of nuclear β-catenin along the domain marked by a yellow box in (D) reveals a nuclear concentration gradient spanning from the center to the periphery of each focus.

(F) Mesp2-GFP mouse reporter line expression shows activation of *Mesp2* in the periphery of synchronized foci.

(G) Foci are identified using immunostaining for β-catenin for the same sample shown in (F).

highly synchronized (Figures 4A and 4B) and calculation of oscillation phases in several foci showed that these were synchronized in phase (Figure 4C).

In vivo, oscillation dynamics and synchronization are complex, leading to signaling activity wave patterns that sweep through the PSM in posterior to anterior direction. These wave patterns are due to both frequency and phase gradients within the PSM (Gomez et al., 2008; Oates et al., 2012). Hence, cells in the posterior PSM oscillate faster than cells located in the more anterior PSM. We analyzed if a frequency gradient is also found in self-organized foci. To this end, we quantified frequencies in space and time within ePSM and indeed found a frequency gradient within oscillating foci, spanning from focus center to the periphery (Figures 4D–4F).

To quantitatively compare the frequency gradient in ePSM to that along the A/P axis of intact mouse PSM, we first directly measured oscillation frequencies within intact mouse PSM and also in 2D segmentation ex vivo assays using real-time imaging (Figure S3). These measurements revealed that, indeed, a similar (but not identical, see below) range of frequencies re-emerges in ePSM and, hence, in drastically reduced spatial dimensions, compared to the frequency gradient found in vivo (Figures 4E and 4F). The frequency gradient in ePSM builds up over time as cells in the periphery of each ePSM, but not in its center, progressively slow down oscillations (Figure 4G). This is again reminiscent of the dynamics that we quantified in vivo within the anterior PSM or in the periphery of 2D ex vivo cultures (Figures 4G and S3). Accordingly, we found that, while at the center of the ePSM foci, the period remained stable at around 150 min during the culture, and while at a distance of 60–70 μm away from the center, the period started from a similar value but progressively increased throughout the culture time (Figure 5G).

Importantly, we found that a frequency gradient is also established when only very posterior tail bud cells with very similar frequency are used for re-aggregation, confirming that the frequency gradient emerges de novo during self-organization (Figure S4). Combined, our findings show that the fundamental dynamic properties of in vivo PSM are fully recapitulated in ePSM and originate in a self-organized manner.

Collective Phase Results from Active Synchronization of PSM Cells

Real-time imaging showed that, before the frequency gradient is established, cells within the ePSM first synchronously oscillate in phase for several cycles. This in-phase rhythm is surprising given that the cells represent a randomly distributed assembly of oscillators with different initial phases (and frequencies, see below). To address how in-phase synchronization is established and how the collective phase is determined, we designed an experimental approach that enables controlled input of phases. To generate cell aggregates with defined phase distributions, it was crucial to separate each individual genetic oscillator (i.e., the PSM cells) according to its phase (Figure 5A). We used fluorescence-activated cell sorting (FACS) of PSM cells carrying the LuVeLu reporter to sort cells based on peak or trough intensity values (Figures 5A, 5B, and S5). Using intensity values as approximation for the state of oscillation phase, the sorted populations, i.e., peak and trough, differ in oscillation phase by half a

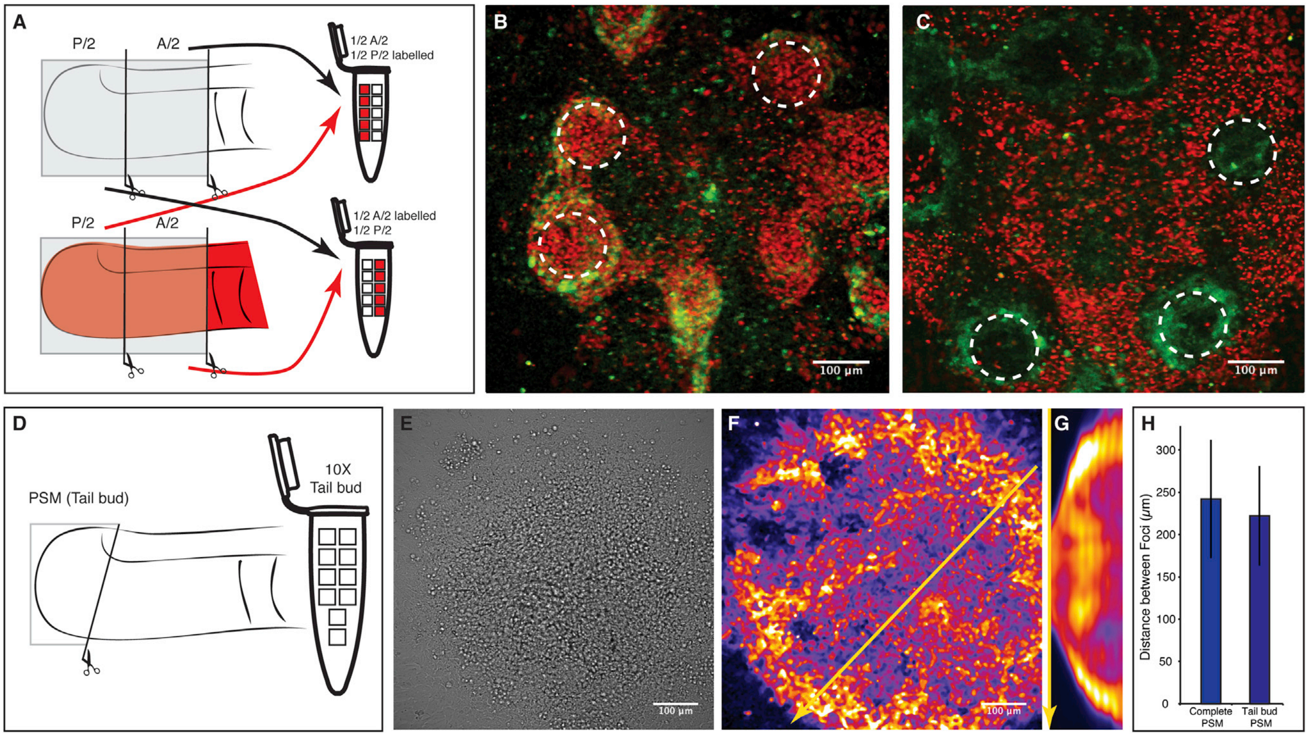

Figure 3. Cell Sorting Takes Place during Spatial Self-Organization, but Initial A/P Differences Are Not Required within the Randomized Cell Population

(A) Schematic of the experimental design. To assess cell sorting based on A/P origin of cells, these were labeled genetically with H2B-mCherry. Labeled cells from posterior PSM are mixed with unlabeled anterior PSM cells, or vice versa. All cells are positive for the LuVeLu reporter. This enabled the assessment of the distribution of anterior and posterior PSM cells in newly formed foci after self-organization.

(B) Posterior PSM cells labeled with H2B-mCherry. After ~22 hr of culture, posterior cells are mostly present in the center of the foci.

(C) Anterior PSM cells labeled with H2B-mCherry. Anterior PSM cells are largely excluded from the centers of the foci.

(D) To address the requirement of sorting, only cells from a much-defined posterior PSM/tail bud identity were used for randomization. These cells share a very similar A/P identity, while anterior PSM cells are missing.

(E) Brightfield view of randomization assay using only tail bud cells.

(F) LuVeLu readout reveals the formation of multiple oscillating foci after randomization of only tail bud cells (same sample shown in E).

(G) Kymograph along the yellow arrow (F) demonstrates coordinated oscillatory LuVeLu reporter activity.

(H) Spatial distance between foci is unaffected by using only posterior tail bud cells for randomization (distance between all PSM cells is 242 ± 70μm [± SD, n = 93] versus distance between foci when only posterior tail bud cells are used, 221 ± 62μm [± SD, n = 49]).

cycle, i.e., π. These sorted cell populations were used separately in self-organization assays and the phase and frequency quantified using real-time imaging experiments.

Strikingly, sorted populations of either peak or trough intensity values reached collective oscillations that occurred in anti-phase, i.e., π-shifted, from each other (Figures 5C and 5D). This indicates that cells retained phase information after dissociation and FACS sorting. This clearly excludes any global oscillator resetting as an underlying cause for synchronization. Rather, as cells are able to retain phase-memory even after dissociation, it shows that active synchronization between initially randomized genetic oscillators is required to achieve complete in-phase oscillations.

More generally, this suggests that the collective, emerging phase of coupled genetic oscillators reflects and depends on the distribution of phases in the original mixture used to start the experiment. This predicts that even physically separated ePSM would exhibit an identical oscillation rhythm, as long as these ePSM are initiated from the same pool of randomized cells. Indeed, we observed that cell aggregates generated from the same cell mixture, but cultured in physical separation from each other, showed foci that oscillated in synchrony (Figure 5E). This demonstrates that the collective rhythm is specified by the properties of the input cell population.

Collective Frequency Is Determined by Integration of All Individual Frequencies

Establishing collective in-phase synchronization requires that PSM cells oscillate with a common frequency. If this is not the case, in-phase oscillations will progressively slip out of phase (Pikovsky et al., 2003). However, at the time of randomization and formation of cell aggregates, input cells not only differ in oscillation phase, but also in respect to their frequency, as they originate from distinct locations along the frequency gradient within the PSM (Figures 5A and S3). The question then arises of how a stable, collective frequency is determined. One

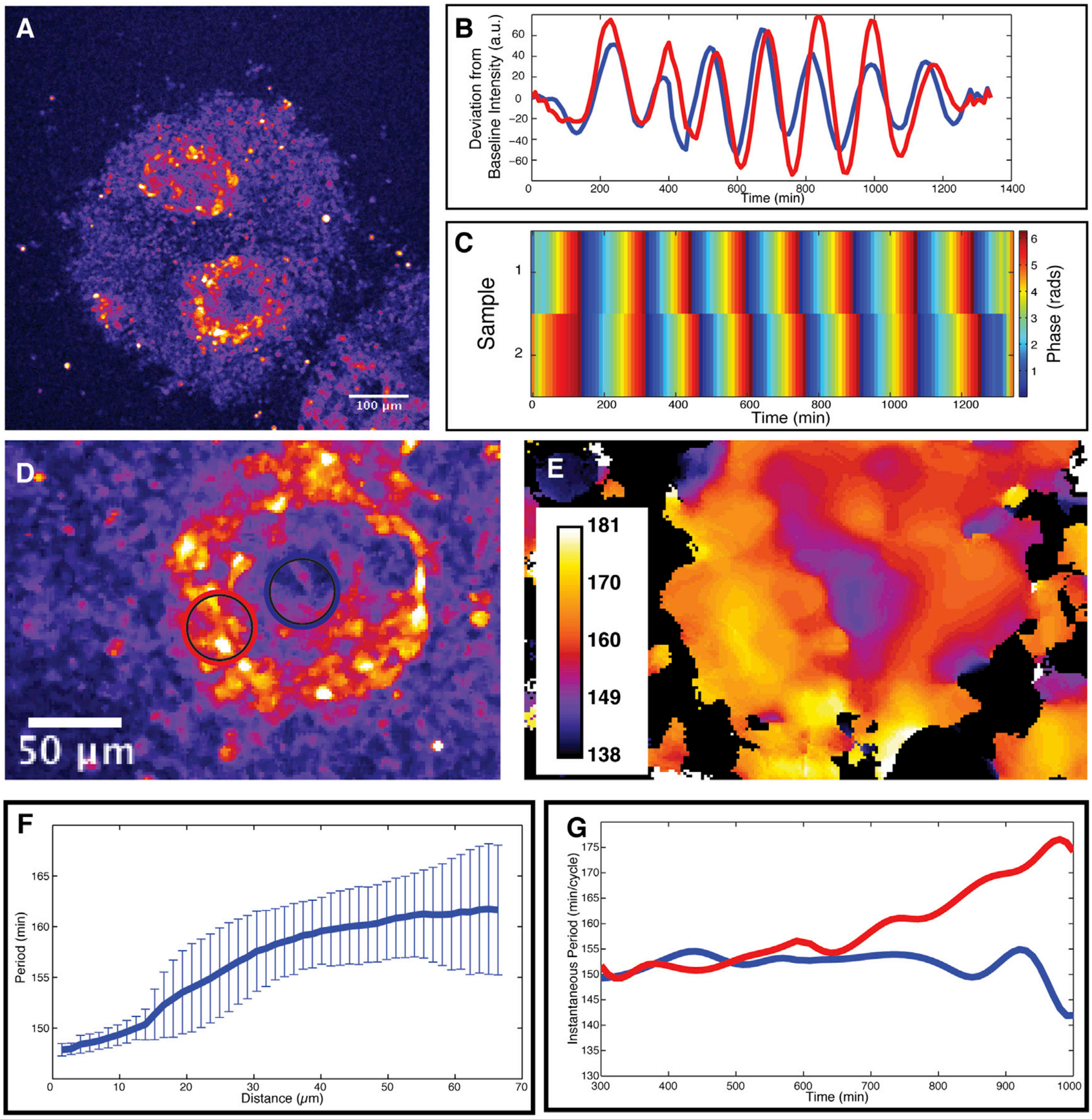

Figure 4. Synchronized Oscillations in ePSM and De Novo Formation of Frequency Gradients

(A) Oscillating ePSM after ~22 hr of culture visualized using LuVeLu reporter expression.

(B and C) Intensity fluctuations (B) and oscillation phase calculations (C) at the center of the two ePSM shown in (A) demonstrate that foci oscillate in phase.

(D and E) Magnification of single ePSM (D) and Fourier transform to calculate oscillation periods for every spatial point within ePSM (E). The period values are color-coded as indicated.

(F) Quantification of spatial period distribution in ePSM (E) reveals period gradient from center to the periphery (± SD, n=36 measurements per ePSM location).

(G) Temporal evolution of period in ePSM center (blue circle in D) compared to periphery (red circle in D). At the center of each ePSM, a stable period over time is recorded (blue), while at the periphery of the focus, the period is increasing over time (red).

possibility is that the collective frequency depends on the input cell population in a similar way as has been found for phase synchronization (Baker and Schnell, 2009; Kuramoto, 2003). Alternatively, if a pacemaker was present, it might enforce its pace on other oscillators, hence, collective frequency would match the frequency of the pacemaker (Pikovsky et al., 2003). To address these distinct possibilities, we compared input and emerging collective frequencies of re-aggregated PSM cells. We exploited the possibility of controlling the distribution of frequencies in the population of input cells by using defined quantities of cells from posterior (i.e., faster oscillators) or anterior (i.e., slower oscillators) PSM. Using this strategy, we performed titration experiments, in which the ratio between anterior and posterior cells was systematically altered (Figure 6A). Since we quantified the spatial distribution of oscillation periods along the PSM in vivo (Figure S3) and the input cell population was known for each titration experiment, we were able to calculate the average period of the input population before

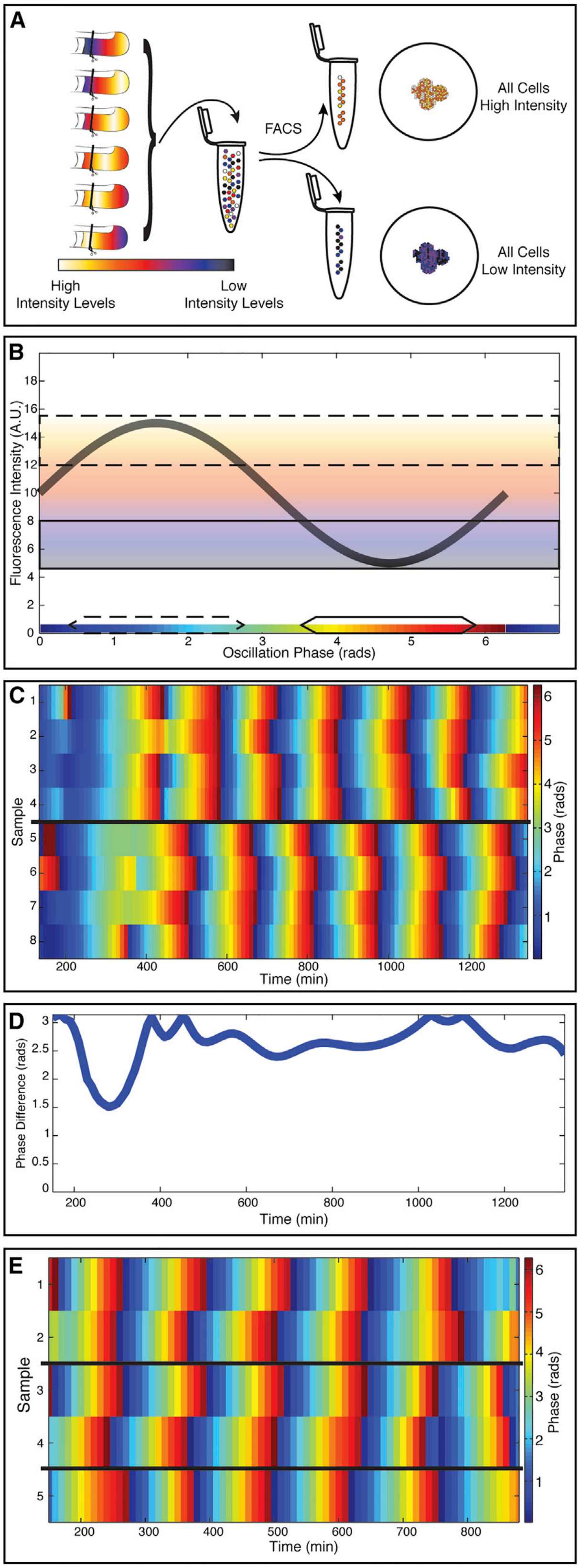

Figure 5. Collective Phase after Randomization Results from Active Synchronization of PSM Cells

(A) FACS sorting is used to separate high (yellow) from low (blue) LuVeLu-expressing cells, approximating cells being close to peak or trough of oscillations, respectively. These distinct cell populations collected from several embryos are then used to initiate separate randomization assays.

(B) Scheme illustrating FACS sorting strategy. PSM cells with high LuVeLu expression (corresponding oscillation phase indicated with the dashed box) are separated from cells at trough LuVeLu expression (oscillation phase indicated with solid box). These sorted populations differ by half oscillation cycle, i.e., π rad.

(C) Oscillation phase representation of foci originating from sorted pools of cells, i.e., either peak (foci 1–4) or trough cells (foci 5–8). Within sorted pools of cells, we found in-phase synchronization within both groups, i.e., within peak or trough pools. In contrast, between peak and trough pools, the corresponding foci show oscillations that occur out of phase, i.e., with a phase shift of π rad. This indicates that phase information is retained after dissociation.

(D) After quantification of phase difference between peak- and trough-sorted cells during self-organization assay, the value remains very close to π. The two populations are oscillating in anti-phase.

(E) Using one randomized cell population (containing all phases, no FACS sorting) to perform several separated randomization experiments simultaneously resulted in ePSM that are in-phase synchronized, and this also occurred if assays were physically separated. Samples 1 and 2, samples 3 and 4, and sample 5 have been cultured in three different, physically separated wells.

synchronization and compare this to the collective period that we measured experimentally after synchronization.

Our results clearly demonstrate that in all titration experiments, cells synchronized and showed in phase, collective oscillations (Figure 6B). By controlling the composition of input cells, we were able to tune the collective period, which remained close to the arithmetic average of the input periods during the titration experiment (Figure 6C). Increasing the fraction of posterior cells, i.e., adding faster oscillators, led to faster oscillations of the entire, in-phase synchronized cell ensemble (Figures 6B and 6C). Hence, while the period of input cells ranged from 120–180 min, a balanced mixture (i.e., 50% P/2 cells; see Figure 6) showed a collective period of 150 min after synchronization. These findings provide clear evidence of collective behavior, and hence, we conclude that cellular oscillation dynamics reflect the integration of the ensemble of oscillators.

Cell Tracking Demonstrates that PSM Cells Change Their Oscillation Dynamics Depending on Surrounding Cell Ensemble

The finding of a collective period after synchronization approximating the arithmetic average of periods in the ensemble of input cells suggests that PSM cells adjust their oscillation dynamics, i.e., either accelerate or slow down oscillations, in response to and depending on the characteristics of their neighbors.

To directly test whether PSM cells change their oscillation dynamics as a function of surrounding cells, we tracked genetically labeled anterior and posterior PSM cells and compared their periods before and after synchronization (Figure 7). Our real-time quantifications demonstrated that, after re-aggregation, originally faster posterior PSM cells and slower anterior PSM cells oscillate in synchrony and share a collective period (Figures 7D and 7E), again matching the arithmetic average of input periods. Importantly, while quantification of oscillations in posterior cells

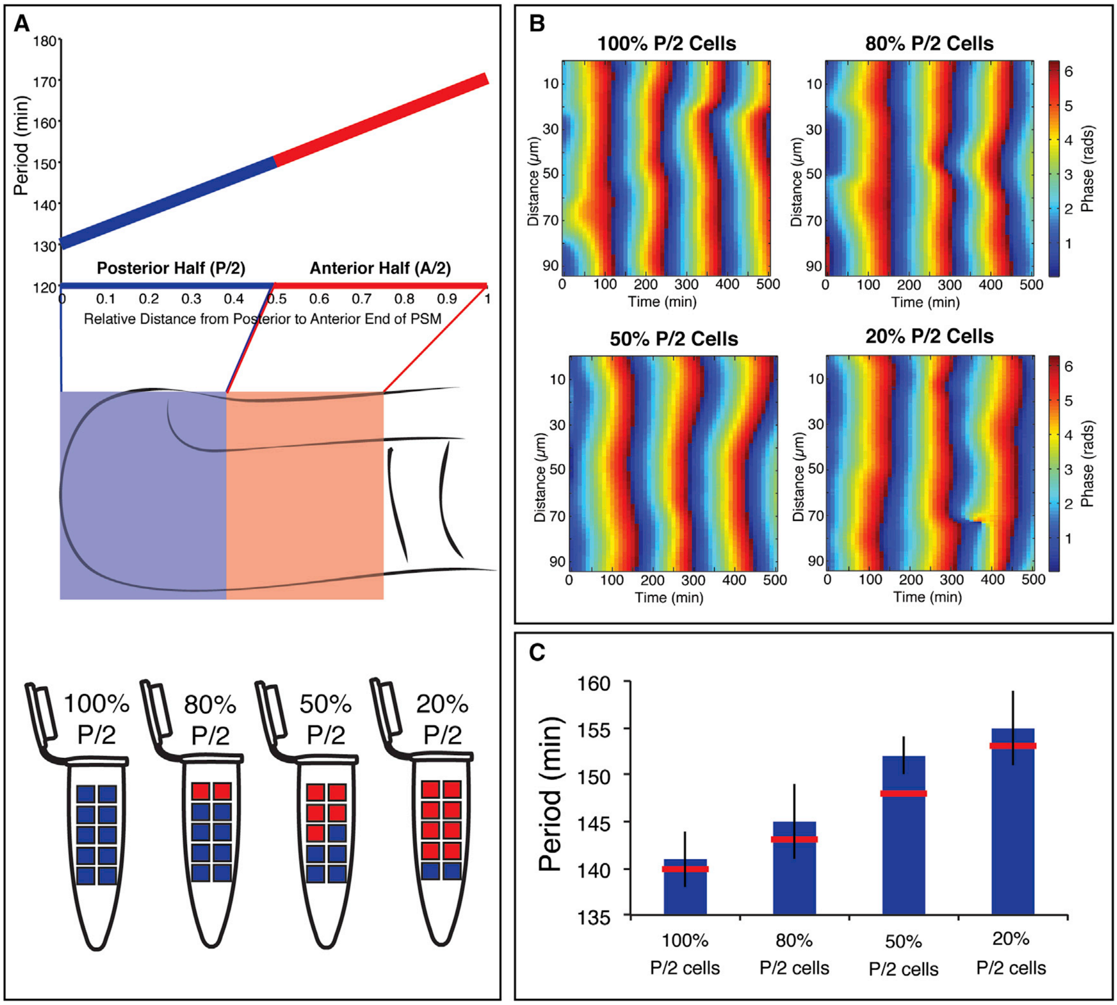

Figure 6. Titration Experiments Show that the Collective Period in ePSM Depends on the Ensemble of Input Cells

(A) Schematic of the experimental design, building on the quantified period gradient along the A/P axis of the PSM. Fast-oscillating cells originating from the posterior-half PSM (P/2, region marked with blue) are mixed at various ratios with slower oscillating cells from the anterior-half PSM (A/2, region marked with red). In these titration experiments, cell ensembles consisting of 100%, 80%, 50%, and 20% P/2 cells are generated and the input periods are known.

(B) Phase kymographs of re-aggregation experiments using cell ensembles with different cell composition shown in (A). During the first hours of culture, oscillations occur synchronized in re-aggregated cells (quantified along 90 μm), independent of cell composition. Note, however, that the collective oscillation period differs according to cell composition (quantified in C).

(C) Quantification of collective period after synchronization in cell ensembles containing 100%, 80%, 50%, and 20% P/2 cells. The measured periods of 141 ± 3 min, 145 ± 4 min, 152 ± 2 min, and 155 ± 2 min (± SEM, n > = 3), respectively, are in close agreement with the predicted value, based on the calculated arithmetic mean of input periods (shown in red). Calculated values are 140 min, 143 min, 148 min, and 153 min, respectively.

revealed that their oscillations slowed down, cell-tracking of anterior PSM cells showed that they oscillate faster after synchronization (Figure 7F). Combined, these findings demonstrate that PSM cells tune their oscillation dynamics as a function of surrounding PSM cells.

DISCUSSION

Here, we have presented evidence for self-organization of PSM cells from disordered initial conditions. When PSM cells are dissociated to single cells and re-aggregated randomly for in vitro culture, coherent spatiotemporal wave patterns form de novo. Within each re-aggregate, wave patterns emerge in multiple foci, which correspond to miniature PSM-structures and which we therefore termed ePSM. The correspondence of ePSM to the in vivo PSM pattern is evident at the level of spatial gene and protein gradient expression patterns. In addition, the dynamics of gene activity oscillations within ePSM match those found in PSM in vivo. We found that cells acquire a collective frequency that depends on the ensemble of cells in the re-aggregate. Accordingly, we were able to tune the collective frequency by performing titration experiments using defined input of fast and slow oscillating cells. Crucially, cell-tracking shows that PSM cells synchronize by accelerating or decelerating their

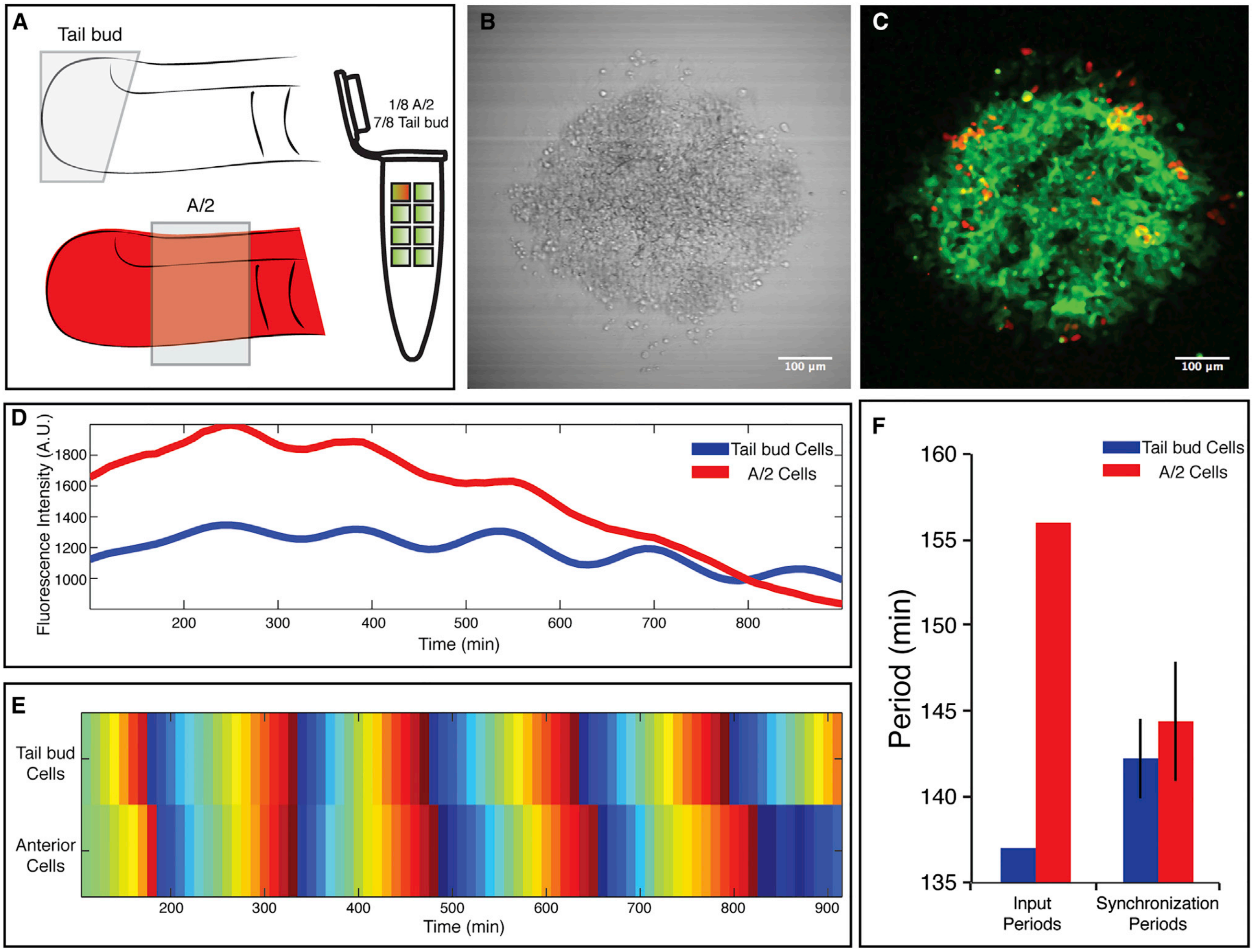

Figure 7. Tracking of PSM Cells Directly Demonstrates Tuning of Cellular Oscillation Dynamics during Synchronization

(A) Schematic of experimental design to track genetically labeled (i.e., H2B-mCherry positive) cells from the anterior half of the PSM (domain boxed in red embryo labeled "A/2"). Within the dissociated cell population used to initiate the experiment, labeled A/2 cells constitute only approximately one-eighth of all cells. The remaining cells are unlabeled and were dissociated from the posterior end of the PSM (tail bud), an area boxed in the unlabeled embryo as "Tail bud"). All cells express LuVeLu-reporter, enabling quantification of oscillations. Using the H2B-mCherry label, cell tracking of anterior PSM cells during synchronization was performed.

(B and C) Bright field (B) and fluorescent (C) image of randomized PSM cells after 500 min of culture, consisting of tail bud cells with small proportion (~12%) of labeled anterior PSM cells.

(D) LuVeLu reporter quantifications in labeled anterior PSM cells (red) and in tail bud cells (blue) reveal synchronized oscillations in both populations.

(E) Phase calculation confirms that after re-aggregation and synchronization, anterior PSM and tail bud cells oscillate synchronized in phase.

(F) Oscillation period in anterior PSM and tail bud cells before randomization differs significantly (period in anterior PSM cells, ~156 min; period in tail bud cells ~137 min; see also Figures 6 and S3). However, after synchronization, direct measurement of oscillation periods in both populations demonstrates that these oscillate with a common collective period, measured for anterior cells to be 144.4 ± 3.5 min and for tail bud cells to be 142.2 ± 2.4 min (± SD, n = 6). Synchronization is achieved by slowing down of the tail bud cells and acceleration of the anterior PSM cells' oscillations. The new collective period reflects the average of all input oscillation periods.

oscillations, depending on the surrounding cells. This provides evidence that oscillation dynamics reflect integration at the system level, i.e., the cell ensemble feeds back on the lower level unit, the individual PSM cells.

While spatial self-organization is accompanied by cell sorting, we provide evidence that initial differences in A/P axial levels, and hence, adhesion properties (Duband et al., 1987), are per se not required to initiate self-organization (Figure 3). In addition, we demonstrate that synchronization of PSM cells involves the tuning of individual cell frequencies to reach a common rhythm (Figures 5–7), a finding that cannot be explained by cell sorting alone.

Several findings regarding the spatial self-organization of ePSM are remarkable. Cells with graded levels of adhesion molecules are known to sort themselves within aggregates (Foty and Steinberg, 2005), with apparent similarity to the separation of

immiscible fluids (Beysens et al., 2000). In these cases, cells with a common property end up together in one cluster (Townes and Holtfreter, 1955). In contrast, in each aggregate of PSM cells, multiple clusters, i.e., synchronized foci, form. Furthermore, the spatial distribution of these foci displays a regularity, which is invariant upon several experimental manipulations, such as a change in input cell population (Figure 3) or perturbation of Notch signaling (Figure S6). Upon Notch-signaling inhibition, cells continue to oscillate without obtaining synchrony, yet, the distance between foci is unaffected compared to control experiments. The robust, macroscopic spatial arrangement of foci indicates the existence of an intrinsic length scale, commonly found in reaction-diffusion patterning systems (Gierer and Meinhardt, 1972; Turing, 1952).

Self-organization is not only reflected at the level of formation of ePSM and their spatial arrangement, but also manifests prominently at the level of temporal coherent oscillatory patterns. How do collective synchronization and oscillation foci emerge? One possibility is the existence of a limited number of specialized cells, i.e., pacemaker cells, which could serve as seeds for synchronization and foci formation. Such pacemaker cells could originate from the posterior PSM, where cells show highest oscillation frequencies. In addition, the posterior PSM contains long-term progenitors, which show stem cell properties (i.e., "axial stem cells") (Cambray and Wilson, 2002, 2007). Indeed, we do observe that cells from the posterior PSM preferentially end up in foci centers, while anterior PSM cells populate the periphery of ePSM. However, several of our results indicate that such a scenario, in which pacemaker cells guide synchronization, does not apply. First, we find that foci are not formed randomly in space, which would be expected by randomized location of pacemaker cells (Figure S1). Second, the number of foci did not increase when only cells from the posterior PSM are used for re-aggregation, which would increase the number of potential pacemaker cells within the cell aggregate (Figure S7). Finally, we found that the collective frequency after synchronization corresponds to the arithmetic average of the input oscillator frequencies, rather than matching the highest frequency of potential pacemaker cells from the posterior PSM (Figure 6). Combined, these findings argue for system-level regulation of ePSM formation and demonstrate that a dependence on pacemaker cells is not evident.

Collective synchronization of PSM cells reveals that the ensemble of genetic PSM oscillators exhibits fundamental characteristics of weakly coupled, phase-oscillator networks (Kuramoto, 2003; Pikovsky et al., 2003). Building on the Kuramoto (2003) model for oscillators coupled via the mean-field, several models have been proposed to account for the observed signaling dynamics during somite segmentation (Baker and Schnell, 2009; Morelli et al., 2009; Murray et al., 2013; Riedel-Kruse et al., 2007). Predictions based on these models were successfully validated at the level of morphological somite formation (Herrgen et al., 2010; Oates et al., 2012). However, direct evidence for collective synchronization as proposed in the Kuramoto (2003) model, at the level of molecular oscillations, has not been achieved so far with PSM cells. Here, we provide, to the best of our knowledge, the first direct, quantitative, and dynamic data showing that PSM cells exhibit collective synchronization and reach an average frequency, as predicted in models for coupled phase oscillators. Future work will build on this experimental system and will decipher the details of the underlying phase-coupling mechanism, including the influence of coupling delays (Morelli et al., 2009).

More generally, while the oscillations seen in our system reflect feedback loops at the level of gene expression regulation and involve a plethora of molecular species and reactions (Hirata et al., 2002; Lewis, 2003), their behavior during synchronization can fundamentally be compared to other examples of collective synchronization, from the coordinated hand clapping of excited human audiences (Néda et al., 2000) to the synchronization of circadian pacemaker cells (Liu et al., 1997), glycolytic oscillations in yeast populations (Weber et al., 2012), and periodically flashing fireflies (Mirollo and Strogatz, 1990). At the same time, the findings of emerging target-wave patterns and an intrinsic length scale underlying the spatial ePSM distribution reveal similarities to excitable media, such as the well-characterized Belousov-Zhabotinsky (BZ) chemical reaction (Ross et al., 1988; Tinsley et al., 2009; Zaikin and Zhabotinsky, 1970) and *Dictyostelium discoideum* aggregation waves (Höfer et al., 1995; Sawai et al., 2007). The system of coupled PSM oscillators therefore exhibits features of several classes of self-organizing systems, such as excitable media and coupled oscillator networks (Dörfler et al., 2013; Goldbeter, 1997; Kuramoto, 2003).

Our results also have implications for the study of signaling dynamics during in vivo embryonic mesoderm development. Signaling dynamics in the PSM generate periodic gene activity waves that are commonly considered to be kinematic in nature (Palmeirim et al., 1997). In this view, kinematic waves reflect the activity of autonomous cellular oscillators and the presence of a spatial frequency gradient (Ross et al., 1988) and, accordingly, do not require any cell-cell communication. Indeed, experimental evidence indicated that physical separation of PSM into tissue fragments does not disrupt wave patterns (Lauschke et al., 2013; Maroto et al., 2005; Masamizu et al., 2006) and that isolated single cells exhibit oscillatory activities (Masamizu et al., 2006). However, we show that wave patterns and frequency gradients form de novo in a self-organized manner, which is incompatible with a purely kinematic wave model. Hence, these wave patterns fundamentally represent a higher-order phenomenon and are based on the integration of oscillator properties within the cell ensemble.

In vivo, the potential for self-organization could be particularly relevant as the first coherent wave patterns emerge when mesoderm is formed during gastrulation. It is conceivable that signaling cues, environmental context, and spatial boundary conditions further restrain self-organization potential in vivo, providing biases that ensure robust outcomes of intrinsically stochastic, self-organized pattern formation. Future studies will need to address these specific aspects of self-organization during the early stages of development and, hence, at the onset of wave patterns in vivo.

In summary, our findings provide a tractable, genetic example for self-organization, collective synchronization, and more generally, for the formation of order from disorder.

EXPERIMENTAL PROCEDURES

Embryo PSM Culture and Randomization Assays

Embryo PSM culture and 2D ex vivo assays were performed as previously described (Aulehla et al., 2008; Lauschke et al., 2013). For randomization assays, entire PSM were isolated and pooled in groups of six. Pooled PSM were gently pipetted to achieve mechanical dissociation of cells. The cells were then filtered through a 10 μm filter (ParTec). In order to obtain randomized PSM cell populations, dissociated cells were centrifuged at 400 rcf for 4 min, and hereby, a cell pellet was formed. Subsequently, this cell pellet was cut in four to five smaller pieces that were plated on fibronectin-coated slides containing culture medium (Lauschke et al., 2013). They were cultured up to 24 hr at 37°C and 5% CO_2. Details on mouse reporter lines are described in Supplementary Experimental Procedures. Animals are housed in the European Molecular Biology Laboratory (EMBL) animal facilities under veterinarian supervision and the guidelines of the European Commission, revised directive 2010/63/EU, and AVMA guidelines 2007.

In Situ Hybridization and Immunofluorescence

In situ hybridization and immunofluorescence on cultured re-aggregated PSM cells was performed as previously described (Aulehla et al., 2008; Lauschke et al., 2013). To quantify nuclear β-catenin levels, nuclei stained with DAPI were segmented using Fiji.

Imaging and Image Processing

Imaging and processing of the data were performed as previously described (Lauschke et al., 2013). Kymographs were generated along the indicated lines using Fiji software. Instantaneous oscillation phases were calculated using the Hilbert transform (Pikovsky et al., 2003).

Quantifications

The distance between neighboring foci was measured using Fiji after their centers were manually marked.

To quantify oscillations, regions of interest (ROIs) were defined in regions within the samples and the signal was processed using Fiji as described (Lauschke et al., 2013). The extracted phase of the oscillations was used to calculate the instantaneous period and amplitude of the signal. Fourier transform on the signal was performed with MATLAB.

To quantify the period gradient along the A/P axis of cultured tails, Fourier transform was performed using real-time LuVeLu imaging quantifications along the entire PSM. Segmentation of H2B-mCherry positive cells was done in Fiji and allowed quantification of LuVeLu oscillations in defined mCherry positive or negative cell populations, respectively.

SUPPLEMENTAL INFORMATION

Supplemental Information includes Supplemental Experimental Procedures, seven figures, and five movies and can be found with this article online at http://dx.doi.org/10.1016/j.cell.2016.01.028.

AUTHOR CONTRIBUTIONS

Conceptualization, A.A. and C.D.T.; Investigation, C.D.T. and A.A.; Writing – Original Draft, C.D.T. and A.A.; Writing – Review & Editing, A.A. and C.D.T.; Supervision, A.A.

ACKNOWLEDGMENTS

We thank Marcus Heisler, Takashi Hiiragi, Francois Spitz, and Aulehla lab members for discussions and comments on the manuscript. This work was supported by EMBL Imaging, Flow Cytometry Core Facility (FACS), and laboratory animal resource core facilities. We thank Bernd Klaus for help in statistical analysis and Alexis Perez Gonzales for FACS analysis. We thank Henning Falk for generation of the graphical abstract. This work received funding from the European Research Council under an ERC starting grant agreement n. 639343 to A.A.

Received: March 23, 2015
Revised: November 20, 2015
Accepted: January 20, 2016
Published: February 11, 2016

REFERENCES

Aulehla, A., Wiegraebe, W., Baubet, V., Wahl, M.B., Deng, C., Taketo, M., Lewandoski, M., and Pourquié, O. (2008). A β-catenin gradient links the clock and wavefront systems in mouse embryo segmentation. Nat. Cell Biol. *10*, 186–193.

Baker, R.E., and Schnell, S. (2009). How can mathematics help us explore vertebrate segmentation? HFSP J. *3*, 1–5.

Beysens, D.A., Forgacs, G., and Glazier, J.A. (2000). Cell sorting is analogous to phase ordering in fluids. Proc. Natl. Acad. Sci. USA *97*, 9467–9471.

Buck, J., and Buck, E. (1966). Biology of Synchronous Flashing of Fireflies. Nature *211*, 562–564.

Camazine, S. (2003). Self-organization in Biological Systems (Princeton University Press).

Cambray, N., and Wilson, V. (2002). Axial progenitors with extensive potency are localised to the mouse chordoneural hinge. Development *129*, 4855–4866.

Cambray, N., and Wilson, V. (2007). Two distinct sources for a population of maturing axial progenitors. Development *134*, 2829–2840.

Delaune, E.A., François, P., Shih, N.P., and Amacher, S.L. (2012). Single-cell-resolution imaging of the impact of Notch signaling and mitosis on segmentation clock dynamics. Dev. Cell *23*, 995–1005.

Dequéant, M.-L., Glynn, E., Gaudenz, K., Wahl, M., Chen, J., Mushegian, A., and Pourquié, O. (2006). A complex oscillating network of signaling genes underlies the mouse segmentation clock. Science *314*, 1595–1598.

Dörfler, F., Chertkov, M., and Bullo, F. (2013). Synchronization in complex oscillator networks and smart grids. Proc. Natl. Acad. Sci. USA *110*, 2005–2010.

Duband, J.L., Dufour, S., Hatta, K., Takeichi, M., Edelman, G.M., and Thiery, J.P. (1987). Adhesion molecules during somitogenesis in the avian embryo. J. Cell Biol. *104*, 1361–1374.

Foty, R.A., and Steinberg, M.S. (2005). The differential adhesion hypothesis: a direct evaluation. Dev. Biol. *278*, 255–263.

Gierer, A., and Meinhardt, H. (1972). A theory of biological pattern formation. Kybernetik *12*, 30–39.

Gierer, A., Berking, S., Bode, H., David, C.N., Flick, K., Hansmann, G., Schaller, H., and Trenkner, E. (1972). Regeneration of hydra from reaggregated cells. Nat. New Biol. *239*, 98–101.

Goldbeter, A. (1997). Biochemical Oscillations and Cellular Rhythms (Cambridge University Press).

Gomez, C., Ozbudak, E.M., Wunderlich, J., Baumann, D., Lewis, J., and Pourquié, O. (2008). Control of segment number in vertebrate embryos. Nature *454*, 335–339.

Herrgen, L., Ares, S., Morelli, L.G., Schröter, C., Jülicher, F., and Oates, A.C. (2010). Intercellular coupling regulates the period of the segmentation clock. Curr. Biol. *20*, 1244–1253.

Hirata, H., Yoshiura, S., Ohtsuka, T., Bessho, Y., Harada, T., Yoshikawa, K., and Kageyama, R. (2002). Oscillatory expression of the bHLH factor Hes1 regulated by a negative feedback loop. Science *298*, 840–843.

Höfer, T., Sherratt, J.A., and Maini, P.K. (1995). Dictyostelium discoideum: cellular self-organization in an excitable biological medium. Proc. Biol. Sci. *259*, 249–257.

Horikawa, K., Ishimatsu, K., Yoshimoto, E., Kondo, S., and Takeda, H. (2006). Noise-resistant and synchronized oscillation of the segmentation clock. Nature *441*, 719–723.

Jiang, Y.J., Aerne, B.L., Smithers, L., Haddon, C., Ish-Horowicz, D., and Lewis, J. (2000). Notch signalling and the synchronization of the somite segmentation clock. Nature *408*, 475–479.

Kuramoto, Y. (2003). Chemical Oscillations, Waves, and Turbulence (Courier Dover Publications).

Lauschke, V.M., Tsiairis, C.D., François, P., and Aulehla, A. (2013). Scaling of embryonic patterning based on phase-gradient encoding. Nature *493*, 101–105.

Lewis, J. (2003). Autoinhibition with transcriptional delay: a simple mechanism for the zebrafish somitogenesis oscillator. Curr. Biol. *13*, 1398–1408.

Liu, C., Weaver, D.R., Strogatz, S.H., and Reppert, S.M. (1997). Cellular construction of a circadian clock: period determination in the suprachiasmatic nuclei. Cell *91*, 855–860.

Maroto, M., Dale, J.K., Dequéant, M.-L., Petit, A.-C., and Pourquié, O. (2005). Synchronised cycling gene oscillations in presomitic mesoderm cells require cell-cell contact. Int. J. Dev. Biol. *49*, 309–315.

Masamizu, Y., Ohtsuka, T., Takashima, Y., Nagahara, H., Takenaka, Y., Yoshikawa, K., Okamura, H., and Kageyama, R. (2006). Real-time imaging of the somite segmentation clock: revelation of unstable oscillators in the individual presomitic mesoderm cells. Proc. Natl. Acad. Sci. USA *103*, 1313–1318.

Mirollo, R.E., and Strogatz, S.H. (1990). Synchronization of Pulse-Coupled Biological Oscillators. SIAM J. Appl. Math. *50*, 1645–1662.

Morelli, L.G., Ares, S., Herrgen, L., Schröter, C., Jülicher, F., and Oates, A.C. (2009). Delayed coupling theory of vertebrate segmentation. HFSP J. *3*, 55–66.

Morohashi, Y., Kan, T., Tominari, Y., Fuwa, H., Okamura, Y., Watanabe, N., Sato, C., Natsugari, H., Fukuyama, T., Iwatsubo, T., and Tomita, T. (2006). C-terminal fragment of presenilin is the molecular target of a dipeptidic gamma-secretase-specific inhibitor DAPT (N-[N-(3,5-difluorophenacetyl)-L-alanyl]-S-phenylglycine t-butyl ester). J. Biol. Chem. *281*, 14670–14676.

Murray, P.J., Maini, P.K., and Baker, R.E. (2013). Modelling Delta-Notch perturbations during zebrafish somitogenesis. Dev. Biol. *373*, 407–421.

Néda, Z., Ravasz, E., Brechet, Y., Vicsek, T., and Barabási, A.L. (2000). The sound of many hands clapping. Nature *403*, 849–850.

Oates, A.C., Morelli, L.G., and Ares, S. (2012). Patterning embryos with oscillations: structure, function and dynamics of the vertebrate segmentation clock. Development *139*, 625–639.

Oginuma, M., Takahashi, Y., Kitajima, S., Kiso, M., Kanno, J., Kimura, A., and Saga, Y. (2010). The oscillation of Notch activation, but not its boundary, is required for somite border formation and rostral-caudal patterning within a somite. Development *137*, 1515–1522.

Okubo, Y., Sugawara, T., Abe-Koduka, N., Kanno, J., Kimura, A., and Saga, Y. (2012). Lfng regulates the synchronized oscillation of the mouse segmentation clock via trans-repression of Notch signalling. Nat. Commun. *3*, 1141–1149.

Palmeirim, I., Henrique, D., Ish-Horowicz, D., and Pourquié, O. (1997). Avian hairy gene expression identifies a molecular clock linked to vertebrate segmentation and somitogenesis. Cell *91*, 639–648.

Pikovsky, A., Rosenblum, M., and Kurths, J. (2003). Synchronization: A Universal Concept in Nonlinear Sciences (Cambridge Nonlinear Science Series) (Cambridge University Press).

Riedel-Kruse, I.H., Müller, C., and Oates, A.C. (2007). Synchrony dynamics during initiation, failure, and rescue of the segmentation clock. Science *317*, 1911–1915.

Ross, J., Müller, S.C., and Vidal, C. (1988). Chemical waves. Science *240*, 460–465.

Saga, Y., Hata, N., Koseki, H., and Taketo, M.M. (1997). Mesp2: a novel mouse gene expressed in the presegmented mesoderm and essential for segmentation initiation. Genes Dev. *11*, 1827–1839.

Sawai, S., Guan, X.-J., Kuspa, A., and Cox, E.C. (2007). High-throughput analysis of spatio-temporal dynamics in Dictyostelium. Genome Biol. *8*, R144.

Stauber, M., Sachidanandan, C., Morgenstern, C., and Ish-Horowicz, D. (2009). Differential axial requirements for lunatic fringe and Hes7 transcription during mouse somitogenesis. PLoS ONE *4*, e7996.

Tinsley, M.R., Taylor, A.F., Huang, Z., and Showalter, K. (2009). Emergence of collective behavior in groups of excitable catalyst-loaded particles: spatiotemporal dynamical quorum sensing. Phys. Rev. Lett. *102*, 158301.

Townes, P.L., and Holtfreter, J. (1955). Directed movements and selective adhesion of embryonic amphibian cells. J. Exp. Zool. *128*, 53–120.

Turing, A.M. (1952). The Chemical Basis of Morphogenesis. Philos. Trans. R. Soc. Lond. B Biol. Sci. *237*, 37–72.

Weber, A., Prokazov, Y., Zuschratter, W., and Hauser, M.J.B. (2012). Desynchronisation of glycolytic oscillations in yeast cell populations. PLoS ONE *7*, e43276.

Yamaguchi, T.P., Takada, S., Yoshikawa, Y., Wu, N., and McMahon, A.P. (1999). T (Brachyury) is a direct target of Wnt3a during paraxial mesoderm specification. Genes Dev. *13*, 3185–3190.

Zaikin, A.N., and Zhabotinsky, A.M. (1970). Concentration wave propagation in two-dimensional liquid-phase self-oscillating system. Nature *225*, 535–537.

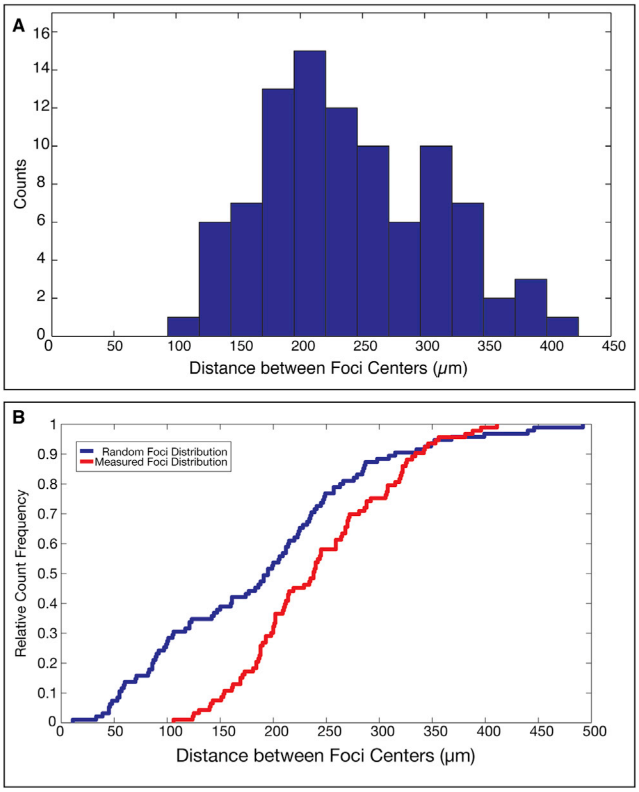

Figure S1. The Spatial Arrangement of Foci Is Not Random, Related to Figure 1F

(A) Histogram of distances between foci. The minimum distance between foci is 100μm.

(B) Cumulative probability distribution of the experimental distance between neighboring foci is significantly different compared to a simulated random one (p value = 4.3*10-5, Kolmogorov-Smirnov test). The random distribution was created in MATLAB. The same number of foci was randomly allocated in areas of similar size and with same foci density as found in PSM cell re-aggregates.

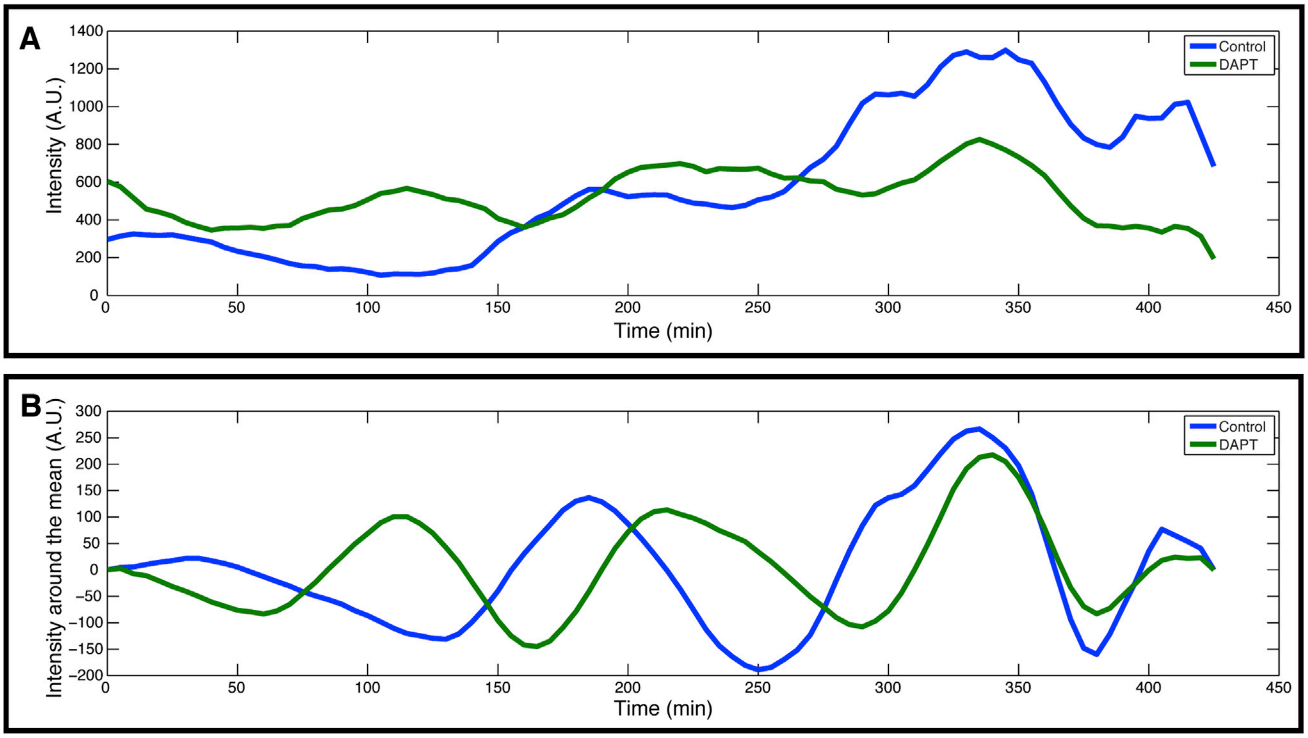

Figure S2. Individual Cells Display Oscillatory Expression of the LuVeLu Transgene in the Presence of Notch Signaling Inhibitor, 2μm DAPT, Related to Figure 1H

Representatives of 7 tracked cells.

(A) Quantification of LuVeLu intensity over time in single cells in control (blue) and DAPT treated samples (green).

(B) LuVeLu signal after subtraction of background trend-line (calculated as moving average) reveals single cells oscillations in both control and DAPT treatment conditions.

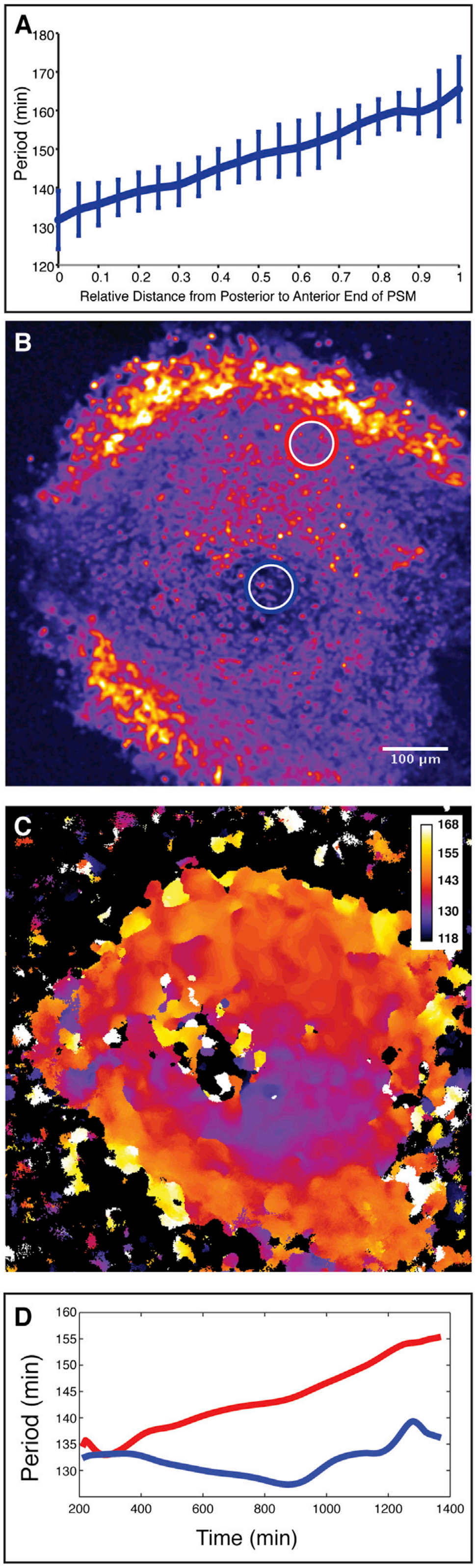

(legend on next page)

Figure S3. Quantification of Frequency Gradients, Related to Figure 4

(A) Quantification of oscillation period along the anterior-posterior axis in intact PSM (mean of 8 different cultured PSMs (±SD)). The quantifications reveal a period gradient ranging from 130 min at the posterior tip to 170 min at the anterior PSM end.

(B) 2D ex vivo assay from Figures 1(B)-(D) with regions of interest (ROIs) at the center (blue) and the periphery (red) used for quantification of LuVeLu oscillation periods.

(C) Fourier transform based on LuVeLu oscillations reveals dominant period for every spatial point in the 2D ex vivo assay. A gradient of periods appears from center to periphery. Period values are color-coded as indicated.

(D) Oscillation periods at the center (blue line) and periphery (red line) measured in corresponding ROIs in (B) measured during 2D ex vivo culture. The period in the center of the culture is stable around 135 min, while the period at the periphery increases over time.

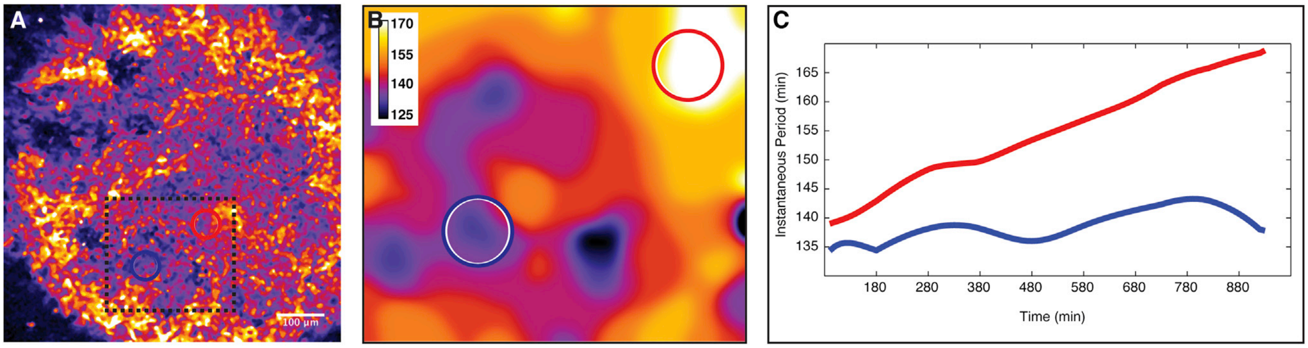

Figure S4. De Novo Formation of Frequency Gradient in Re-aggregates Using Only Tail Bud Cells, Related to Figure 4

(A) Re-aggregate (from Figure 3F) with ROIs within the center (blue ROI) and periphery (red ROI) of a single ePSM (boxed with black dashed line).

(B) Fourier transform based on LuVeLu oscillations in ePSM (black dashed box in A) reveals LuVeLu oscillation periods, that are graded from center to the periphery.

(C) Oscillation period during culture of re-aggregate, measured at the center (blue) and periphery (red) of a single ePSM. Throughout the culture, the period in the center is stable around 135 min, while at the periphery the period increases over time, leading to a frequency gradient.

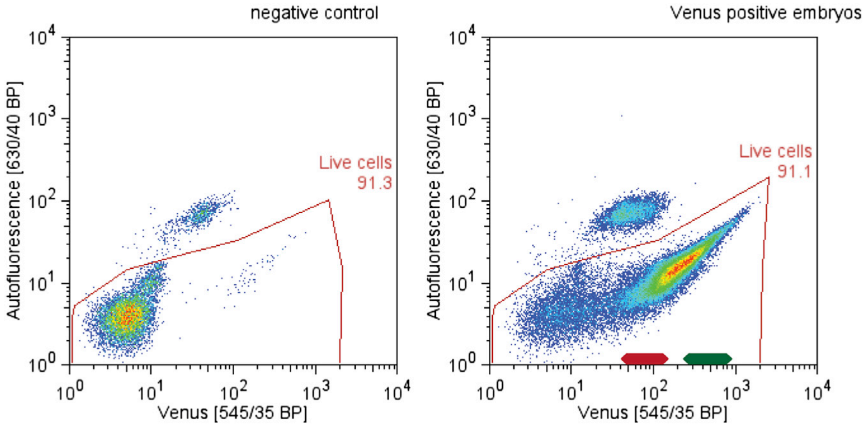

Figure S5. FACS Allows the Identification of Cell Populations with High and Low Venus Expression in PSM Cells of LuVeLu Mice, Related to Figure 5.

(Left) To determine background fluorescence PSM cells of control (LuVeLu negative) embryos were used for FACS analysis. Few LuVeLu positive cells were spiked into the cell mixture to identify the expected dynamic range.

(Right) FACS analysis using PSM of LuVeLu transgenic embryos permits the collection of separate cell populations expressing low and high levels of Venus, respectively (indicated by red and green lines).

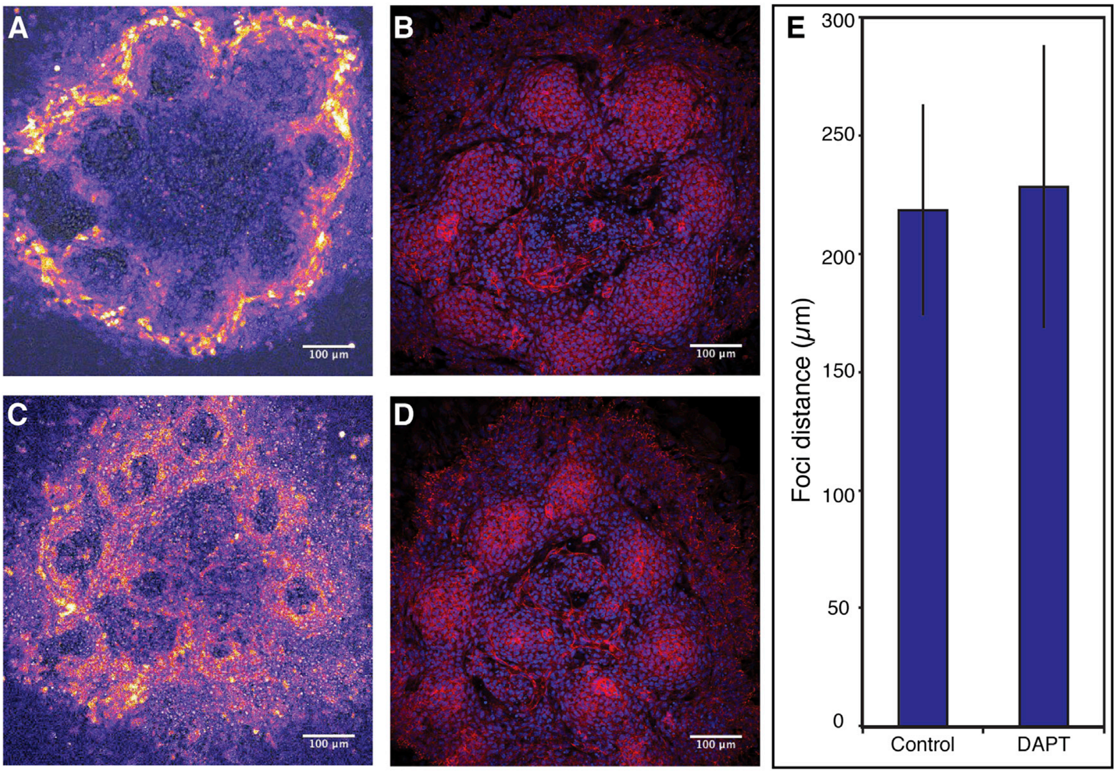

Figure S6. Effect of Notch Signaling Inhibition Using DAPT on Spatial Patterning in Re-aggregation Assay, Related to Figure 1H

(A) Randomization assay using dissociated and re-aggregated PSM cells. Periodic LuVeLu expression waves appear in multiple foci within the culture.

(B) Immunostaining against β-catenin (red) in sample shown in A. Foci are marked by a peak of nuclear β-catenin. Nuclei are labeled using DAPI (blue).

(C and D) Randomization assay with DAPT (2µM) treatment. Spatial patterning is visible at the level of LuVeLu foci (C) and immunostaining against β-catenin (D).

(E) Analysis of average distance between foci in control (219 ± 45µm SD, n = 23) and for the DAPT treated samples (228 ± 60µm SD, n = 19) indicates no change of spatial foci arrangement upon Notch-inhibition.

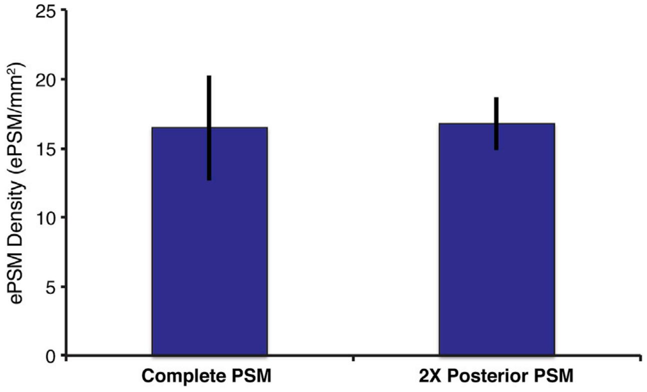

Figure S7. The Density of ePSM Remains Constant Independent of the Input Cell Population, Related to Figure 6
The comparison shown is between re-aggregates using cells from the entire PSM (16.5 ± 3.8 foci/mm^2, ± SD, n = 4) versus re-aggregates using only cells from the posterior PSM halves (16.8 ± 1.9 foci/mm^2, ± SD, n = 4). To compensate for cell numbers, twice as many posterior PSM halves were used.

Cell, Volume *164*

Supplemental Information

Self-Organization of Embryonic Genetic Oscillators into Spatiotemporal Wave Patterns

Charisios D. Tsiairis and Alexander Aulehla

Supplemental Experimental Procedures

Culture and Imaging Conditions

Chambers slides (Lab-Tek) were coated with 50 μg/ml fibronectin in PBS as previously described (Lauschke et al., 2013). The tails of E10.5 mouse embryos were dissected in HEPES-buffered culture medium (DMEM-F12, Cell Culture Technologies, with addition of 0.5 mM glucose, 2 mM glutamine and 1% BSA). Depending on the experimental needs the PSM was cut in halves along the A/P axis, or only the tip of the PSM was excised (Lauschke et al., 2013).The pieces of PSM were combined in groups and dissociated by gentle pipetting. Cells were re-aggregated by centrifugation at room temperature, 400 rcf for 4 min. The pellet of cells was collected and cut in pieces approximately 250μm X 250μm.

When the cell origin was tracked the PSM tissue of E10.5 mouse embryos from $LuVeLu^{het}$;$H2BmCherry^{het}$ X $CD1^{wt}$ crosses were used (Abe et al., 2011; Aulehla et al., 2007). Embryos were sorted according to the fluorescence markers and the PSM pieces were isolated as described. Cells were dissociated in groups according to their origin and mixed as experimental needs dictated.

To inhibit Notch signaling reaggregated PSM cells from the entire PSM were cultured as previously described with culture medium supplemented with 2 μM DAPT (Sigma-Aldrich) (Morohashi et al., 2006).

Imaging was performed with a Zeiss LSM780 laser-scanning microscope. Depending on the recorded fluorophore, reaggregated PSM cells were excited with a Ti:Sapphire Laser (Chameleon-Ultra, Coherent) at a wavelength of 960nm and/or a DPSS 561nm Laser through a 20X Plan Apo objective (numerical aperture 0.8) . A Z-stack of 3–4 planes at a 3-5 μm distance was collected every 10 min.

Mouse strains

The mice used were of the previously described lines R26-H2B-mCherry, LuVeLu, and Mesp2-GFP (Abe et al., 2011; Aulehla et al., 2007; Morimoto et al., 2006). We obtained the Mesp2-GFP line from RIKEN BRC through the National Bio-Resource Project of the MEXT, Japan.

FACS Sorting

For sorting according to the intensity, the dissociated cells were sorted using a MoFlo Legacy High Speed cell sorter (Beckman Coulter Inc.) equipped with a 100 μm nozzle. BD FACSFlow™ sheath (Becton Dickinson GmbH), filtered in-line through a PALL Fluorodyne II filter O.2μm (Pall GmbH), was used as sheath in the acquisition of the samples. Acquisition was triggered on FSC using a

512/15 bandpass (BP) filter and the same filter was used for SSC measurements. Venus fluorescence intensity was measured after filtering through a 545/35 nm bandpass filter, while a second detector measured cellular autofluorescence through a 630/40 nm BP filter. Data was acquired using MoFlo Summit software (Beckman Coulter), while post-acquisition analysis was done with FlowJo 9.2 software (Tree Star, Inc). The sorting was completed within 30 min and the sorted cells were processed as previously described to form aggregates.

Data and Image Analysis

The distance between neighbor foci was measured in FIJI, and MATLAB was used to generate their distribution as well as the comparison to random distribution with the Kolmogorov-Smirnov test. Comparison of mean interfoci distance between different experimental groups was done with Student's t-test in Excel (Microsoft).

The phases of the oscillation in the selected region of interest (ROI) were calculate using the Hilbert transform of the signal as previously described (Lauschke et al., 2013). The phases were unwrapped and differentiated using MATLAB to calculate instantaneous frequency whose inverse is the instantaneous period. The absolute values of the complex numbers generated by Hilbert transform correspond to the collective amplitude inside the specified ROI (Pikovsky et al., 2003). This was normalized to the initial value of the time series to generate a measure of the synchronization inside the ROI.

To compare the phase difference in the signal of different ROIs the extracted phases were subtracted as angles.

Fourier transform was performed using MATLAB for the cosine of the phase signal to identify the dominant frequency in this ROI. The ROI was reduced to a single pixel and Fourier transform was performed for each of them when a spatial map of dominant periods was generated for the sample.

References

Abe, T., Kiyonari, H., Shioi, G., Inoue, K.-I., Nakao, K., Aizawa, S., and Fujimori, T. (2011). Establishment of conditional reporter mouse lines at ROSA26 locus for live cell imaging. Genesis *49*, 579–590.

Aulehla, A., Wiegraebe, W., Baubet, V., Wahl, M.B., Deng, C.-X., Taketo, M.M., Lewandoski, M., and Pourquié, O. (2007). A β-catenin gradient links the clock and wavefront systems in mouse embryo segmentation. Nat Cell Biol *10*, 186–193.

Lauschke, V.M., Tsiairis, C.D., Francois, P., and Aulehla, A. (2013). Scaling of embryonic patterning based on phase-gradient encoding. Nature *493*, 101–105.

Morimoto, M., Kiso, M., Sasaki, N., and Saga, Y. (2006). Cooperative Mesp activity is required for normal somitogenesis along the anterior-posterior axis. Dev. Biol.

300, 687–698.

Morohashi, Y., Kan, T., Tominari, Y., Fuwa, H., Okamura, Y., Watanabe, N., Sato, C., Natsugari, H., Fukuyama, T., Iwatsubo, T., et al. (2006). C-terminal fragment of presenilin is the molecular target of a dipeptidic gamma-secretase-specific inhibitor DAPT (N-[N-(3,5-difluorophenacetyl)-L-alanyl]-S-phenylglycine t-butyl ester). J. Biol. Chem. *281*, 14670–14676.

Pikovsky, A., Rosenblum, M., and Kurths, J. (2003). Synchronization: A Universal Concept in Nonlinear Sciences (Cambridge Nonlinear Science Series) (Cambridge University Press).
